# Targeting the initiator to activate both ferroptosis and cuproptosis for breast cancer treatment: progress and possibility for clinical application

**DOI:** 10.3389/fphar.2024.1493188

**Published:** 2025-01-10

**Authors:** Murshid Imam, Jiale Ji, Zhijie Zhang, Shunchao Yan

**Affiliations:** Department of Oncology, Shengjing Hospital of China Medical University, Shenyang, China

**Keywords:** ferroptosis, cuproptosis, breast cancer, interplay, immune infiltration, prognosis, cross talk

## Abstract

Breast cancer is the most commonly diagnosed cancer worldwide. Metal metabolism is pivotal for regulating cell fate and drug sensitivity in breast cancer. Iron and copper are essential metal ions critical for maintaining cellular function. The accumulation of iron and copper ions triggers distinct cell death pathways, known as ferroptosis and cuproptosis, respectively. Ferroptosis is characterized by iron-dependent lipid peroxidation, while cuproptosis involves copper-induced oxidative stress. They are increasingly recognized as promising targets for the development of anticancer drugs. Recently, compelling evidence demonstrated that the interplay between ferroptosis and cuproptosis plays a crucial role in regulating breast cancer progression. This review elucidates the converging pathways of ferroptosis and cuproptosis in breast cancer. Moreover, we examined the value of genes associated with ferroptosis and cuproptosis in the clinical diagnosis and treatment of breast cancer, mainly outlining the potential for a co-targeting approach. Lastly, we delve into the current challenges and limitations of this strategy. In general, this review offers an overview of the interaction between ferroptosis and cuproptosis in breast cancer, offering valuable perspectives for further research and clinical treatment.

## 1 Introduction

Breast cancer is the most common cancer among women worldwide (Kciuk et al., 2024). The incidence of breast cancer is increasing each year, and there will be more than 3 million new breast cancer cases annually by 2040 (Arnold et al., 2022). Despite advances in treatments such as surgery, chemotherapy, radiotherapy, targeted therapy, and endocrine therapy (Nagini, 2017), patients still face the risk of recurrence and mortality, with 685,000 deaths recorded in 2020 (Courtney et al., 2022). Consequently, it is urgent to identify novel biomarkers to predict the biological malignancy of tumors and support the development of targeted therapies.

Metal ions are crucial nutrients for living organisms (Jomova et al., 2022), serving as cofactors in nucleic acid and protein functions and playing critical roles in respiration, metabolism, and biosynthesis (Chang, 2015; Witkowska et al., 2021). Maintaining metal homeostasis chiefly depends on precisely regulating metal uptake, distribution, and excretion (Aron et al., 2015; Yang et al., 2024b). Iron, an essential trace transition metal, is indispensable for the functioning of living organisms (Liang and Ferrara, 2020). It plays pivotal roles in oxygen transportation, cell respiration, energy generation, and DNA synthesis (Abbasi et al., 2021). In breast cancer biology, iron has emerged as a critical element, that significantly influences tumor initiation, progression, and response to treatment through mechanisms such as estrogen redox cycling and oxidative stress (Ali et al., 2024; Scimeca and Bonanno, 2018; Torti and Torti, 2013). Maintaining appropriate iron levels is crucial (Anderson and Frazer, 2017) because excessive iron accumulation could be toxic (Bogdan et al., 2016; Ge et al., 2024; Kohgo et al., 2008; Liang and Ferrara, 2020). This toxicity could lead to ferroptosis, a type of cell death driven by iron-dependent lipid peroxidation (Angeli et al., 2014; Rishi et al., 2021; Zarjou et al., 2013). Copper, an essential trace element, plays a critical role in various metabolic processes and is implicated in breast cancer progression due to its involvement in cellular signaling pathways (Ge et al., 2022; Ramchandani et al., 2021; Yang L. et al., 2023). Similar to iron, the regulation of copper levels within human cells is rigorously controlled, as deviations from the optimal concentration impair biological processes and trigger cell death. Perturbations in copper homeostasis could lead to cuproptosis (Li L. et al., 2024), which explicitly affects mitochondrial lipoylated proteins (Tang et al., 2022; Wang et al., 2023b).

Ferroptosis and cuproptosis have garnered significant attention because of their unique features and distinctive regulatory mechanisms (Chen T et al., 2024). Recently, emerging evidence has suggested an intriguing convergence between ferroptosis and cuproptosis, revealing shared regulatory mechanisms (Liu and Chen, 2024; Wang J et al., 2024). In light of this, we aimed to present and explore the intersecting pathways of ferroptosis and cuproptosis within the context of breast cancer. By elucidating these convergences, we endeavor to offer insights that could augment treatment strategies for breast cancer. Concurrently, our review endeavors to assess the genes associated with ferroptosis and cuproptosis in breast cancer, probing their potential as prognostic markers and indicators of treatment response. Additionally, laying the foundation for futuristic opportunities to induce or co-target ferroptosis and cuproptosis holds promise for further enhancing treatment modalities in breast cancer management.

## 2 Converging pathways of ferroptosis and cuproptosis in breast cancer

### 2.1 Dysregulation of metal homeostasis

Metal ions are essential nutrients for living organisms, play critical roles as cofactors in nucleic acid and protein functions, and are indispensable for fundamental biological processes (Chang, 2015). Iron and copper share analogous characteristics, serving as vital nutrients and participating in pivotal biological functions. Their roles are indispensable for sustaining health and are relevant for understanding and managing various diseases (Lippard, 1999; Tsang et al., 2021). Within the framework of breast cancer, iron has emerged as a key player in its onset, progression, and relapse. Its impact spans diverse mechanisms, encompassing oxidative stress induction, DNA damage, estrogen signaling modulation, angiogenesis stimulation, and disruption of intracellular iron metabolism (Chang et al., 2019; Huang, 2008; Islam et al., 2022; Torti and Torti, 2013). Correspondingly, copper demonstrates intricate associations with myriad signaling pathways, thereby exerting a substantial influence on the malignant behavior of breast cancer (Chen L. et al., 2022).

Therefore, maintaining optimal iron and copper levels is crucial for biological functions such as oxygen transport, DNA synthesis, and antioxidant defenses (Bleackley and Macgillivray, 2011; Hirota, 2019; MacDonald, 2000; Prasad, 2014). Acknowledging the dual nature of iron and copper in biological systems is pivotal. While these metals are essential for life at appropriate concentrations, excesses and deficiencies can be detrimental. Excessive iron accumulation, for instance, leads to the formation of a labile iron pool, inducing cellular toxicity and contributing to cellular damage (Schneider and Bhatia, 2013). Similarly, inadequate or excessive copper could be detrimental to organismal growth. An overload of copper can heighten cellular toxicity and oxidative stress, impairing cell proliferation and function (Wang et al., 2023e). This imbalance has been linked to various conditions, including cancer, hematological disorders, brain injury, and other chronic ailments frequently encountered in clinical practice (Schneider and Bhatia, 2013). Researchers have ingeniously leveraged the connection between imbalanced metal levels and cancer progression, effectively transforming the modulation of metal levels into a treatment strategy for combating tumors. This approach has led to significant tumor suppression outcomes (Hunsaker and Franz, 2019).

Metal homeostasis modulation involves three dimensions: the removal of surplus intracellular metal ions, their redistribution across tumor cells, and their accumulation at toxic levels within cancerous cells. New strategies for cancer treatment target metal homeostasis, utilizing nanomolecule-based chelators, ionophores, metal complexes, and metal-based nanomaterials. These approaches regulate the tumor microenvironment, inhibit cell proliferation, and induce cell death (Steinbrueck et al., 2020; Weekley and He, 2017).

Iron and copper are central to diverse cellular death pathways, activating various mechanisms such as ferroptosis, cuproptosis, apoptosis, autophagy, necroptosis, and pyroptosis, each with unique pathways. Recent research highlights iron and copper as inducers of ferroptosis, while cuproptosis is a newly identified cell death mode specifically induced by copper (Hirschhorn and Stockwell, 2019; Tsvetkov et al., 2022). Therefore, manipulating cellular iron and copper homeostasis and targeting metabolic pathways could be potential strategies for treating breast cancer by inducing ferroptosis and cuproptosis. Exploring the convergence of iron and copper homeostasis may provide promising therapeutic avenues for breast cancer treatment.

#### 2.1.1 Dysregulation of iron homeostasis

Iron, an essential micronutrient for biological processes (Forbes, 2009), serves critical functions across various metabolic pathways, such as facilitating oxygen transportation, supporting energy metabolism, aiding nucleotide synthesis, and participating in electron transport. Iron homeostasis, which is critical for fundamental physiological processes, is regulated and maintained by iron metabolism (Hentze et al., 2010; Prá et al., 2012). Maintaining iron levels within a balanced range is crucial, as excessive amounts can lead to toxicity (Hentze et al., 2010). Imbalances in the molecular processes governing iron absorption, utilization, storage, and elimination contribute to disease development (Muñoz et al., 2011a; Muñoz et al., 2011b; Theil, 2000). Excessive iron accumulation, coupled with its propensity to generate reactive oxygen species (ROS), suggests its potential involvement in various chronic illnesses, including diabetes, neurological disorders, cardiomyopathy, and several human cancers, such as lung cancer, colorectal cancer, and breast cancer (Jiang et al., 2004; Menshawey et al., 2020; Sempos et al., 1994; Stevens et al., 1988; Ward and Cloonan, 2019). Iron-induced oxidative stress may damage DNA, proteins, and organelles by producing harmful radicals and hydrogen peroxide via Haber–Weiss and Fenton-type reactions (Eaton and Qian, 2002; Nelson, 1992; Toyokuni, 1996). Furthermore, the role of iron in cell proliferation implies its importance in the expansion of malignant cells and may confer a selective advantage for tumor growth (Marques et al., 2014). In breast cancer cases, dysregulation of iron-binding proteins, such as transferrin, which facilitates the delivery of ferric ions into cells, and iron-transporting proteins, like ferroportin, which is responsible for exporting iron out of cells, is frequently observed. One observed phenomenon involves the downregulation of ferroportin levels by hepcidin through posttranscriptional modifications. This results in a greater ratio of hepcidin to ferroportin, ultimately leading to increased ferritin expression and subsequent iron overload (Pinnix et al., 2010; Zhang et al., 2014). Moreover, alterations in transferrin levels have also been noted (Chang et al., 2019; Rufaida Mustafa Ahmed and Nazik Elmalaika Obaid Seid Ahmed, 2017). Iron overload significantly increases the vulnerability of postmenopausal women to breast cancer development by stimulating and fostering oxidative stress (Huang, 2008; Jian et al., 2011). Moreover, recent research has indicated that elevated iron levels within the inflammatory microenvironment of breast tissue may contribute to the progression and metastasis of breast cancer (Cheng et al., 2020). Increased estrogen levels in breast tissue disturb intracellular iron metabolism, leading to excess iron. This excess iron can trigger the generation of superoxide anions and convert ferritin-bound Fe^3+^ to Fe^2+^, inducing estrogen-induced oxidative stress on nucleic acids and subsequent breast carcinogenesis (Islam et al., 2022). Therefore, directing interventions toward iron metabolic pathways and modulating cellular iron homeostasis could present a promising avenue for breast cancer treatment, by utilizing strategies such as iron chelation and addressing iron overload.

**FIGURE 1:**
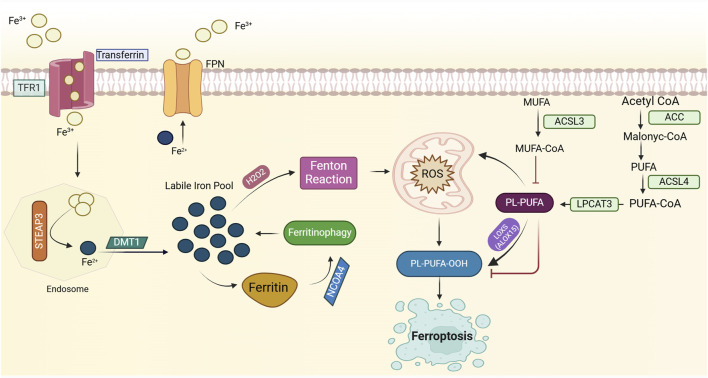
Mechanisms of ferroptosis induction. Ferroptosis is an iron-dependent form of cell death characterized by lipid peroxidation. Elevated intracellular iron, imported via transferrin (TF) and transferrin receptor 1 (TFR1), is reduced to its ferrous form (Fe^2^⁺) by STEAP3, and subsequently transported to the labile iron pool by DMT1. This labile iron contributes to the generation of reactive oxygen species (ROS), particularly hydroxyl radicals (⋅OH), through the Fenton reaction, where Fe^2^⁺ catalyzes the conversion of hydrogen peroxide (H₂O₂) into highly reactive ROS. ROS, in turn, initiate oxidative damage, including the peroxidation of polyunsaturated fatty acid phospholipids (PUFA-PLs), which serve as key substrates for ferroptosis. Enzymes such as acetyl-CoA carboxylase (ACC), long-chain acyl-CoA synthetase 4 (ACSL4), and lysophosphatidylcholine acyltransferase 3 (LPCAT3) facilitate the incorporation of PUFAs into phospholipids, thereby promoting lipid peroxidation. Ferroptosis is further exacerbated by ferritinophagy, a process that releases iron from ferritin stores, thereby increasing ROS production through NCOA4-mediated ferritin degradation. Lipid peroxidation is driven by iron-dependent lipoxygenases, producing toxic phospholipid hydroperoxides (PUFA-PL-OOH). Cellular sensitivity to ferroptosis is regulated by the dynamic balance of iron import/export (e.g., TFR1, ferroportin (FPN)), iron storage (e.g., ferritin), and lipid metabolism. Please see the text for specific details. Fe^3+^, ferric ion; Fe^2+^, ferrous ion; TFR1, transferrin receptor 1; FPN, ferroportin; STEAP3, six transmembrane epithelial antigen of prostate 3; H2O2, hydrogen peroxide; ROS, reactive oxygen species; PL-PUFA-OOH, phospholipid polyunsaturated fatty acid hydroperoxide, NCOA4, Nuclear Receptor Coactivator 4; ROS, Reactive Oxygen Species; MUFA, Monounsaturated Fatty Acids; ACSL3, Acyl-CoA Synthetase Long-Chain Family Member 3; MUFA-CoA, Monounsaturated Fatty Acid Coenzyme A; PL-PUFA, Phospholipid Polyunsaturated Fatty Acids; LOXS, Lipoxygenases; PL-PUFA-OOH, Phospholipid Polyunsaturated Fatty Acid Hydroperoxide; LPCAT3, Lysophosphatidylcholine Acyltransferase 3; PUFA, Polyunsaturated Fatty Acids; ACSL4, Acyl-CoA Synthetase Long-Chain Family Member 4; ACC, Acetyl-CoA Carboxylase.

The excess cellular iron reacts with hydrogen peroxide (H_2_O_2_) in the Fenton reaction, producing harmful hydroxyl radicals. These radicals damage lipids, proteins, and DNA, potentially leading to ferroptosis. Ferroptosis, dependent on iron, involves phospholipid peroxidation resulting from disrupted cellular iron homeostasis and redox balance (Dixon et al., 2012; Jiang X et al., 2021). Recently, researchers have delved deeply into the mechanisms of ferroptosis, concentrating on three main areas: iron metabolism and ROS production, lipid metabolism, and the system Xc-GSH-GPX4 pathway (Hassannia et al., 2019; Jiang X et al., 2021). Elevated iron accumulation is necessary to initiate ferroptosis, and alterations in genes and proteins involved in iron metabolism can modulate ferroptosis sensitivity by modifying cellular iron levels (Jiang X et al., 2021). Transferrin (TF) binds to Fe^3+^ with high affinity in the bloodstream. Once bound to TF, Fe^3+^ is transported into cells via transferrin receptor 1 (TFR1). Inside endosomes, STEAP3 reduces Fe^3+^ to Fe^2+^, which is then transported into the labile iron pool by divalent metal transporter 1 (DMT1). Once in the labile iron pool, Fe^2+^ contributes to the production of ROS and facilitates lipoxygenase activation. Excess iron is stored in ferritin, which undergoes autophagy-mediated degradation, a process defined as ferritinophagy, releasing labile Fe^2+^ and promoting lipid peroxidation (Fang et al., 2023; Jiang X et al., 2021; Paul et al., 2017; Zheng and Conrad, 2020). Furthermore, ROS production is augmented by mitochondrial metabolism (Jiang X et al., 2021; Stockwell, 2022). Importantly, cellular iron import or export is critical for regulating ferroptosis. Transferrin and its receptor (TFR1) enable iron transport into cells, thus initiating ferroptosis (Figure 1) (Wang et al., 2023b). The modulation of ferroptosis involves regulating the expression of TFR1, illustrating its capacity to either promote or inhibit the process. Ferritin heavy chain 1 (FTH1) modulates iron storage by interacting with NCOA4, which initiates iron autophagy. This process releases iron, triggering mitochondrial lipid peroxidation and subsequent ferroptosis (Fang et al., 2021). Research indicates that knocking out NCOA4 diminishes iron autophagy, making cells more resistant to ferroptosis (Fang et al., 2021). Moreover, ferroportin (FPN), an essential iron exporter for maintaining iron balance (Yang et al., 2020), has been identified as a potential target for preventing ferroptosis through the regulation of FPN protein degradation (Traeger et al., 2022; Yang et al., 2020). Hence, modulating cellular sensitivity to ferroptosis through regulating iron metabolism has emerged as an effective strategy for treating ferroptosis-related diseases (Jiang X et al., 2021). Clinically, iron chelators are employed to eliminate excess iron from the body, representing a viable treatment option for managing diseases associated with ferroptosis (Feng W. et al., 2022). Polyunsaturated fatty acid phospholipids (PUFA-PLs) act as key substrates for lipid peroxidation (LPO) (Feng W. et al., 2022), and the biosynthetic pathway of PUFA-PLs is crucial in regulating the initiation of ferroptosis (Feng W. et al., 2022; Li and Li, 2020). Lipid metabolism, one of the three core metabolic pathways, is critical in the development of cardiovascular disease, obesity, cancer, and other conditions (Bacci et al., 2021). As a result, enzymes regulating lipid synthesis, breakdown, and β-oxidation have become prominent therapeutic targets (Zechner et al., 2012). Abnormalities in lipid metabolism, particularly in fatty acid metabolism, are now widely acknowledged as key drivers in the pathogenesis of ferroptosis (Li and Li, 2020; Liang et al., 2022). Monounsaturated fatty acids (MUFAs) and polyunsaturated fatty acids (PUFAs) exert opposing effects on ferroptosis, likely due to their differences in chemical structure and oxidative stability, which influence their susceptibility to lipid peroxidation and thereby modulate ferroptosis (Li and Li, 2020; Magtanong et al., 2019). The balance between MUFA-PLs and PUFA-PLs, derived from monounsaturated and polyunsaturated fatty acids, respectively, is critical in modulating cellular sensitivity to ferroptosis (Li and Li, 2020; Magtanong et al., 2019). In this process, acetyl-CoA is first converted to malonyl-CoA via acetyl-CoA carboxylase (ACC), leading to the production of PUFAs. Long-chain acyl-CoA synthetase 4 (ACSL4) and lysophosphatidylcholine acyltransferase 3 (LPCAT3) (Stockwell, 2022) then facilitate the incorporation of PUFAs into phospholipids (PUFA-PLs) (Stockwell, 2022). Under the action of iron-dependent lipoxygenases and reactive oxygen species (ROS), PUFA-PLs are oxidized into PUFA-PL-OOH, triggering the onset of ferroptosis (Li and Li, 2020; Magtanong et al., 2019). Furthermore, the enzymatic activities of ACSL3 and ACSL4 play a critical role in modulating cellular sensitivity to ferroptosis (Figure 1) (Li and Li, 2020; Magtanong et al., 2019). Interestingly, ACSL3 activation has been found to protect cells from ferroptosis, with studies highlighting its role in gastric and breast cancer cells (Ma et al., 2022; Xie et al., 2022).

#### 2.1.2 Dysregulation of copper homeostasis

Copper, a crucial trace transition metal, is essential for enzymes and proteins in diverse organisms and influences biological processes like mitochondrial function, respiration, antioxidant defense, and cell proliferation. It exists primarily in two oxidation states, Cu^+^ and Cu^2+^, impacting its bioactivity (Ying et al., 2023). Cellular copper homeostasis is tightly regulated by copper-dependent proteins such as copper transporter 1 (CTR1) for uptake, copper chaperones for transport, and copper-transporting P-type ATPases (copper ATPases) for export, ensuring that optimal intracellular copper levels are crucial for overall health. Disruptions in copper homeostasis have been observed in various cancers like colorectal, lung, and breast cancer (Chen L. et al., 2022; Denoyer et al., 2015; Jiang Z et al., 2023). Copper holds significant importance in tumorigenesis and cancer progression, as it can bind and activate key molecules within multiple signaling pathways present in cancer, including breast cancer cells. Within these pathways, the Wnt signaling pathway is vital for maintaining breast cancer stem cells (BCSCs) stemness. Recent studies have suggested that disulfiram/copper (DSF/Cu) complexes can hinder cancer cell proliferation and metastasis by reducing the expression of β-catenin and C-myc, which are critical components of the Wnt pathway (Shivnani et al., 2023; Wang et al., 2020). In addition, the Notch pathway is essential for multiple biological processes in breast cancer cells, encompassing cell differentiation, apoptosis, and cell cycle progression (Farnie and Clarke, 2007). In essence, copper influences breast cancer metastasis through the promotion of Notch ligand shedding and the activation of key signaling pathways, such as the RAS-RAF-MEK-ERK and receptor tyrosine kinase (RTK)-related pathways, ultimately facilitating cancer cell migration and proliferation (Grasso et al., 2021). Furthermore, several studies have shown elevated copper levels in tumor tissues and serum across different cancer types, including breast, gastric, and lung cancers. In breast cancer patients, high serum copper levels are correlated with advanced tumor stage and disease progression (Guo et al., 2021; Heuberger et al., 2022; Jin et al., 2022; Pavithra et al., 2015). These findings underscore the significant regulatory role of copper in breast cancer signaling pathways, emphasizing its importance in treatment strategies. Thus, targeting alterations in copper homeostasis holds promise as a strategy to combat breast cancer.

**FIGURE 2:**
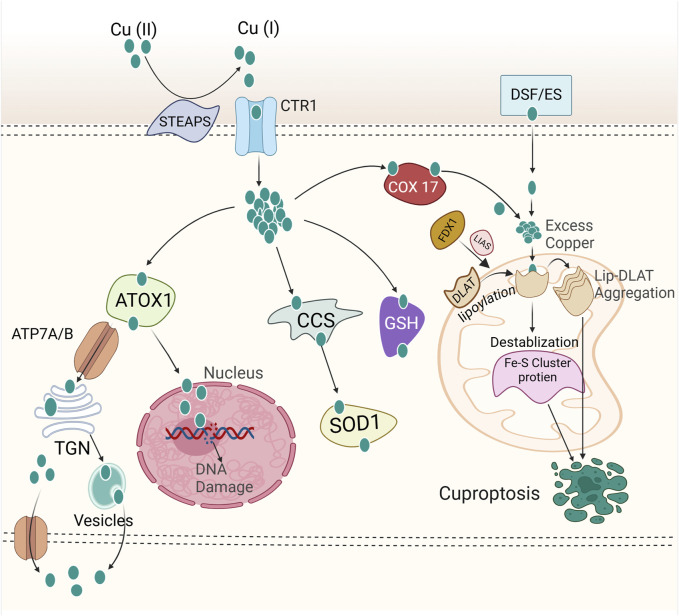
Mechanism of cuproptosis. Copper ions are reduced to their monovalent form (Cu⁺) by STEAP family proteins. Extracellular copper is imported into cells via copper ionophores (e.g., elesclomol) or through copper transporters such as CTR1 and DMT1. Once copper enters the cell, it binds to various copper chaperone proteins, such as ATOX1, CCS, and SOD1, which facilitate its transport to specific subcellular compartments, including the mitochondria, trans-Golgi network (TGN), and nucleus. The nucleus is implicated in cuproptosis through copper’s role in regulating gene expression and DNA repair. Disrupted copper homeostasis induces oxidative stress and DNA damage responses, promoting cellular stress that could lead to cuproptosis. ATP7A and ATP7B or ATP7A/B mediate copper efflux, maintaining intracellular copper homeostasis. Inside the cell, FDX1 reduces Cu^2^⁺ to Cu⁺, which interacts with lipoic acid synthetase (LIAS) to facilitate the lipoylation of key metabolic enzymes, including *DLAT*. It is hypothesized that Cu⁺ directly binds to lipoylated proteins, promoting their oligomerization. This oligomerization results in a toxic gain-of-function, ultimately triggering cell death via cuproptosis. Please see the text for specific details. FDX1, Ferredoxin 1; DLAT, Dihydrolipoamide S-acetyltransferase; CTR1, Copper Transporter 1; COX17, Cytochrome c Oxidase Copper Chaperone 17; DSF/ES, Disulfiram/Elesclomol; STEAPS, Six Transmembrane Epithelial Antigen of Prostate; ATOX1, Antioxidant 1 Copper Chaperone; SOD1, Superoxide Dismutase 1; CCS, Copper Chaperone for Superoxide Dismutase; GSH, Glutathione; TGN, Trans-Golgi network; ATP7A/B, ATPase copper transporting α and ATPase copper transporting β.

Excessive copper can induce the death of breast cancer cells, primarily through a newly identified regulatory cell death mechanism known as cuproptosis (Figure 2) (Tsvetkov et al., 2022; Wang et al., 2023e). Copper uptake into cells is predominantly mediated by copper transporter 1 (CTR1), also known as solute carrier family 31 member 1 (SLC31A1) (Kuo et al., 2001). Cellular copper homeostasis is tightly regulated by adaptive mechanisms that modulate CTR1 expression based on intracellular copper levels. In response to copper depletion, CTR1 expression is upregulated to enhance copper uptake, whereas excess intracellular copper triggers the downregulation of CTR1 to prevent toxicity (Kuo et al., 2012). After entering the intracellular environment, copper is directed to specific subcellular compartments where it is either utilized for essential biochemical processes or sequestered to avoid toxicity. This precise trafficking is mediated by a network of copper chaperone proteins, ensuring its proper distribution and regulation within the cell. Cytochrome c oxidase copper chaperone (COX17) is essential for the delivery of copper to the mitochondrial cytochrome c oxidase complex, a process crucial for sustaining copper homeostasis within the electron transport chain. This copper-dependent mechanism is vital for the proper function of oxidative phosphorylation, enabling efficient ATP production (Palumaa et al., 2004). An additional key mediator in copper distribution is the copper chaperone for superoxide dismutase (CCS), which facilitates the transfer of copper to superoxide dismutase 1 (SOD1). SOD1, a critical antioxidant enzyme, plays a pivotal role in mitigating oxidative stress by catalyzing the dismutation of superoxide radicals, thereby protecting cells from ROS (Wright, 2020). Additionally, the antioxidant 1 copper chaperone (ATOX1) facilitates the transfer of copper to the ATPase copper-transporting α and β (ATP7A and ATP7B or ATP7/B), which are transmembrane proteins responsible for copper efflux. It ensures the regulated export of excess intracellular copper, maintaining copper homeostasis and preventing toxic accumulation (Hatori and Lutsenko, 2016; Schmidt et al., 2018). ATP7A/B primarily reside in the trans-Golgi network (TGN), where they facilitate copper transport from the cytosol into the TGN lumen, ensuring copper homeostasis and delivering it to enzymes in the secretory pathway. Elevated intracellular copper levels induce the translocation of these proteins from the TGN to vesicular compartments, which fuse with the plasma membrane to export copper. Once copper levels return to normal, ATP7A and ATP7B relocate back to the TGN (Hasan et al., 2012). As a result of their critical role in copper transport, mutations in ATP7A and ATP7B disrupt copper homeostasis, leading to Menkes disease and Wilson disease, respectively (de Bie et al., 2007). Proteins regulating copper metabolism interact intricately to maintain intracellular copper homeostasis. However, disruptions in copper homeostasis can result in excessive copper accumulation within cells, potentially leading to cuproptotic cell death. Cancer cells dependent on mitochondrial respiration exhibit increased vulnerability to cuproptosis, with mitochondrial electron transport chain inhibitors suppressing this form of cell death, highlighting the link between cuproptosis and mitochondrial metabolism (Tsvetkov et al., 2022). CRISPR screens have identified key regulators of cuproptosis, with ferredoxin 1 (FDX1) emerging as a central player. FDX1, a target of the copper ionophore elesclomol, reduces Cu^2^⁺ to its more toxic form, Cu⁺, and regulates protein lipoylation through interaction with lipoic acid synthetase (LIAS), which catalyzes the final step of lipoic acid biosynthesis (Figure 2) (Tsvetkov et al., 2022; Tsvetkov et al., 2019). Protein lipoylation, a posttranslational modification, is essential for the function of key enzymes in the mitochondrial tricarboxylic acid (TCA) cycle, including dihydrolipoamide S-acetyltransferase (DLAT), a subunit of the pyruvate dehydrogenase complex (Rowland et al., 2018). Genetic depletion of LIAS and *DLAT*, like FDX1 deletion, confers resistance to cuproptosis (Tsvetkov et al., 2022). Notably, despite their crucial role in cuproptosis, levels of FDX1, LIAS, and protein lipoylation decline during the process, potentially indicating a cellular attempt to counteract excessive copper toxicity by dampening protein lipoylation—an ultimately futile strategy. These findings emphasize the pivotal role of protein lipoylation in cuproptosis. The exact mechanisms by which protein lipoylation triggers cuproptosis remain unclear. However, direct binding of copper to lipoylated *DLAT* has been observed, resulting in protein oligomerization during cuproptosis (Tsvetkov et al., 2022). This suggests that the resultant protein aggregates may induce a toxic gain-of-function effect, ultimately driving cuproptotic cell death (Figure 2). These findings highlight a promising area for future investigation into the complex and distinctive process of cuproptosis.

#### 2.1.3 Convergence between iron and copper homeostasis

Copper complexed with disulfiram has attracted increased amounts of attention due to its anticancer effects (Zha et al., 2014). A recent study demonstrated that DSF/Cu activates ferroptosis in TNBC cells. Treatment with DSF/Cu led to increased lipid peroxidation, upregulation of *HMOX1*, and decreased levels of GPX4 and GSH, ultimately inducing cancer cell death through ferroptosis (Chu et al., 2023). DSF/Cu has emerged as a promising agent for inducing ferroptosis in TNBC cells, offering potential avenues for anticancer therapy (Ren et al., 2021).

The copper homeostasis gene, prion protein *(PRNP),* was notably downregulated in breast cancer cells, correlating with a better prognosis (Lin et al., 2023). *PRNPs* are involved in cancer-related signaling pathways, particularly those governing inflammatory responses and oxidative phosphorylation. *PRNP* overexpression markedly enhanced gefitinib sensitivity in BRCA cells. Overexpression of *PRNP* led to elevated ROS production following gefitinib treatment, while the ferroptosis-selective inhibitor ferrostatin-1 mitigated this increase in ROS levels in BRCA cells. *PRNP* expression was positively correlated with macrophages, Th1 cells, neutrophils, and B cells, while negatively correlated with NK CD56 bright cells and Th17 cells in BRCA. Single-cell analysis showed that *PRNP* was highly expressed in M1 phenotype macrophages, essential tumor-suppressing cells in the tumor stroma. These observations suggest that *PRNP* is potentially involved in ROS-mediated ferroptosis, highlighting its candidacy as a novel therapeutic target for chemotherapy and immunotherapy in breast cancer. Additionally, *PRNP* is correlated with a better prognosis and regulates ferroptosis following gefitinib treatment in breast cancer cells (Lin et al., 2023).

The convergence of iron and copper homeostasis is a promising avenue for inducing ferroptosis and cuproptosis in cancer cells. DSF/Cu and *PRNP* modulation offer potential therapeutic strategies for cancer treatment (Chu et al., 2023; Lin et al., 2023). Future research could focus on elucidating the precise mechanisms underlying the interplay between copper and iron homeostasis in regulating ferroptosis and cuproptosis in cancer cells. Additionally, exploring novel therapeutic agents/complexes that target both ferroptosis and cuproptosis could pave the way for advancements in breast cancer treatment.

### 2.2 Mitochondrial metabolism

**FIGURE 3:**
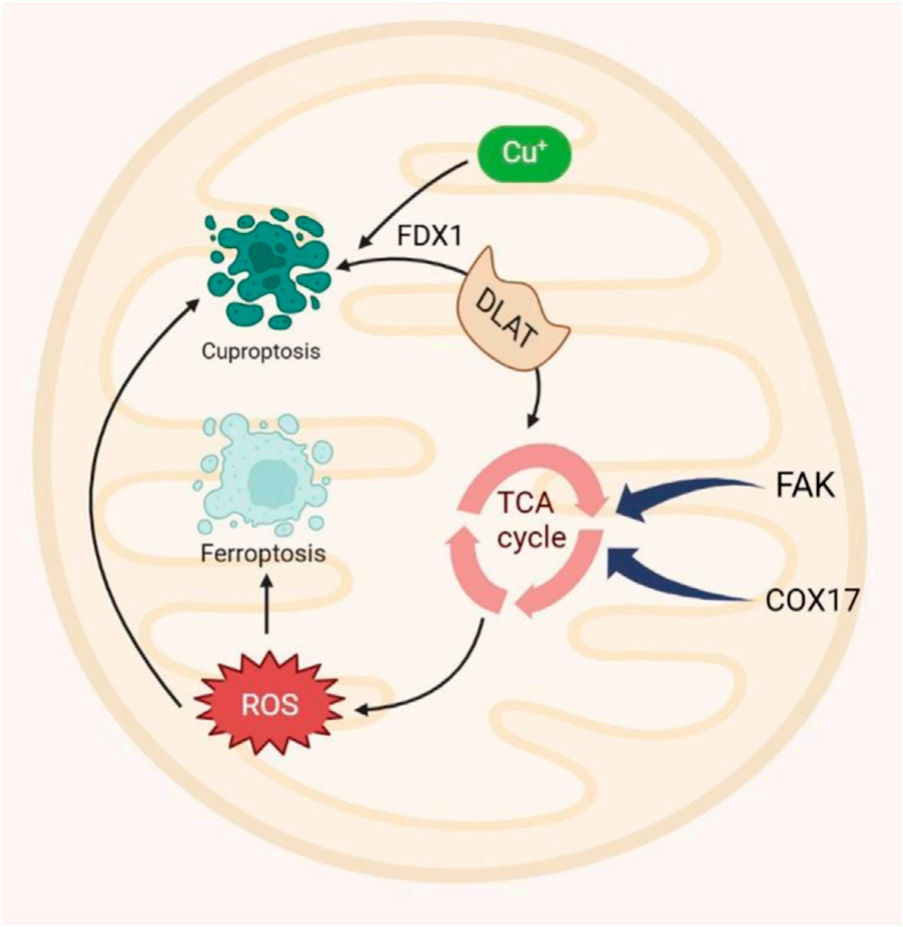
Mitochondria play a central role in ferroptosis by serving as major sources of intracellular ROS. Similarly, cuproptosis occurs in mitochondria, with the TCA cycle acting as a shared pathway for both processes. Dihydrolipoamide S-acetyltransferase (DLAT), a key TCA cycle component, is lipoylated by FDX1, and its interaction with copper triggers cuproptosis. This underscores the mitochondrial TCA cycle as a convergence point for ferroptosis and cuproptosis. Additionally, COX7A1 and FAK accelerate the TCA cycle, promoting the production of metabolites that drive ferroptosis and cuproptosis. ROS, reactive oxygen species; COX7A1, *cytochrome c oxidase 7A1; FAK; focal adhesion kinase. FDX1, ferredoxin 1.*

Mitochondria, crucial for cellular energy production, play vital roles in various physiological and pathological processes (Brand et al., 2013). Mitochondria play a crucial role in the development of numerous human diseases and the regulation of multiple cell death pathways (Javadov et al., 2020). Research has revealed intimate connections between mitochondrial metabolism and ferroptosis, and cuproptosis (Wang J. et al., 2024), highlighting the need to characterize mitochondria for insights into underlying mechanisms, regulatory pathways, and disease ramifications. Mitochondria depend on iron, copper, and calcium for optimal ATP generation. The presence of these metal ions modulates the mitochondrial electron transport chain and other functions, influencing both ferroptosis and cuproptosis (Figure 3). This highlights a potential for anticancer therapy by targeting mitochondrial metabolism to address cuproptosis and ferroptosis simultaneously (Prasad Panda and Kesharwani, 2023). Moreover, the mitochondrial TCA cycle has emerged as a pivotal convergence point for ferroptosis and cuproptosis pathways, further underscoring its importance in governing cellular fate regulation (Liu and Chen, 2024).

#### 2.2.1 Mitochondrial metabolism and ferroptosis in breast cancer

Mitochondria have elevated iron concentrations (Lill and Freibert, 2020). Ferroptosis results in prominent morphological changes and dysfunction in mitochondria (Doll et al., 2017). The interaction between mitochondria and ferroptosis is well-documented, as they jointly regulate iron metabolism and lipid peroxidation (Wang J et al., 2024). While various therapeutic modalities, including endocrine therapy, immunotherapy, radiation, targeted therapy, and chemotherapy, are available for breast cancer treatment (Dong et al., 2024), interventions specifically targeting mitochondria to induce ferroptosis remain underexplored. The recently identified iron-sulfur proteins *CISD1* and *CISD2* facilitate the proliferation of breast cancer cells (Sohn et al., 2013). Silencing *CISD1* and *CISD2* or targeting breast cancer mitochondria with the mitogen derivative MAD-28 can destabilize iron-sulfur proteins, elevate mitochondrial iron accumulation, and hinder breast cancer cell growth (Bai et al., 2015). MAD-28 is highly selective, causing iron accumulation in the mitochondria of breast cancer cells only while leaving normal breast cells unaffected. This selectivity suggests that MAD-28 could be a promising agent for targeting ferroptosis and developing anticancer therapies (Dong et al., 2024). In addition, lipid metabolism, a crucial mitochondrial pathway in ferroptosis, has numerous applications in breast cancer diagnosis and treatment, with a focus on its regulation. Stearoyl-CoA desaturase-1 (SCD1) is upregulated in recurrent human breast cancer samples, indicating a poor prognosis for cancer patients. Thus, the expression of SCD1 can serve as a biomarker for breast cancer recurrence (Luis et al., 2021). Furthermore, Herceptin, also known as trastuzumab, a medication targeting HER-2 in breast cancer treatment, was found to elevate mitochondrial ROS levels in rat cardiomyocytes while reducing GPX4 expression, thereby leading to ferroptosis. The subsequent application of the ferroptosis inhibitor ferrostatin-1 successfully reversed the increase in ROS levels. This observation indicates the potential utility of ferroptosis inhibitors in mitigating cardiotoxicity associated with HER-2-positive breast cancer therapy (Sun L. et al., 2022). Furthermore, manganese superoxide dismutase 2 (SOD2), a key enzyme within the ETC, has emerged as a potential biomarker for breast cancer progression and a target for ferroptosis induction (Sui et al., 2022). Nanomaterial-based drugs, like sorafenib (Louandre et al., 2013) and simvastatin (Yao X. et al., 2021), hold considerable potential for inducing efficient ferroptosis in breast cancer cells without systemic toxicity (Li K. et al., 2022; Sang M et al., 2019; Yao X. et al., 2021).

In conclusion, mitochondria serve as central regulators of ferroptosis in breast cancer, and targeting mitochondrial metabolism represents a promising avenue for inducing ferroptosis in specific breast cancer subtypes. However, further research is warranted to elucidate the precise underlying mechanisms and optimize therapeutic strategies targeting mitochondrial pathways for effective breast cancer treatment.

#### 2.2.2 Mitochondrial metabolism and cuproptosis in breast cancer

Mitochondria are pivotal copper-dependent organelles responsible for energy production through the indispensable cuproenzyme cytochrome c oxidase (CCO) (Carr and Winge, 2003). Disturbances in intracellular copper homeostasis, including loss-of-function mutations in genes crucial for copper regulation, invariably result in lethal genetic disorders such as Wilson disease and Menkes disease (Członkowska et al., 2018; Tümer and Møller, 2010). The mitochondrial TCA cycle is pivotal in the process of cuproptosis, where protein lipoylation is confined to four specific proteins *(DBT, GCSH, DLST, and DLAT)* involved in this cycle. This process is primarily mediated by the mitochondrial carrier family (MCF) (Chen L. et al., 2022; Tang et al., 2022).

Cuproptosis initiates with the accumulation of copper in the cytoplasm and organelles. This prompts the clustering of mitochondrial lipoylated modules and the destabilization of Fe-S cluster proteins essential for mitochondrial function (Chen L. et al., 2022; Tang et al., 2022). Cuproptosis is intricately connected to mitochondrial respiration, and the status of mitochondrial respiratory function impacts its susceptibility (Tang et al., 2022; Yuan et al., 2022). Recently, significant attention has been directed toward understanding the role of mitochondria and cuproptosis in breast cancer (Gururaja Rao, 2017; Liu X. et al., 2023; Porporato et al., 2018). Breast cancer cells undergo metabolic reprogramming, shifting from glycolysis to increased mitochondrial oxidative phosphorylation (OXPHOS) and metabolism, supporting rapid proliferation and metastasis (Li and Li, 2021). Mitochondrial dynamics exhibit flexibility, transitioning between different forms to adapt to microenvironmental changes and therapeutic stress, aiding cancer cell survival (Avagliano et al., 2019). Breast cancer stem cells (CSCs) and circulating tumor cells (CTCs) rely heavily on mitochondrial metabolism and OXPHOS for tumor initiation, metastasis, and treatment resistance (Wedam et al., 2023). Key oncogenes and tumor suppressors, such as *Myc, TP53, PIK3CA*, and Bcl-2 family proteins, regulate mitochondrial metabolism, contributing to tumor progression (Wedam et al., 2023). Mitochondrial dysfunction and alterations in metabolic pathways, such as those involving lipids, amino acids, and the TCA cycle, contribute to drug resistance, suggesting that targeting these pathways could enhance chemotherapy efficacy (Li and Li, 2021; Wedam et al., 2023). Importantly, targeting mitochondrial metabolism through the induction of cuproptosis represents a significant research avenue for cancer therapy, including breast cancer treatment. Breast cancer cells that rely on mitochondrial respiration are particularly susceptible to cuproptosis induction (Ai L. et al., 2024; Xie et al., 2023). Targeting mitochondria to induce cuproptosis in breast cancer cells leverages the role of copper in triggering cell death (Wang J. et al., 2024).

### 2.3 GSH metabolism

GSH plays a pivotal role in both ferroptosis and cuproptosis, serving distinct functions in each pathway (Badgley et al., 2020; Tsvetkov et al., 2022). In ferroptosis, it acts as an antioxidant, inhibiting lipid peroxidation (LPO), while in cuproptosis, it functions as a copper chaperone, binding copper to mitigate the aggregation of lipoylated proteins (Badgley et al., 2020; Tsvetkov et al., 2022). Interestingly, GSH has been shown to have inhibitory effects on both ferroptosis and cuproptosis, suggesting a converging point (Liu and Chen, 2024).

**FIGURE 4:**
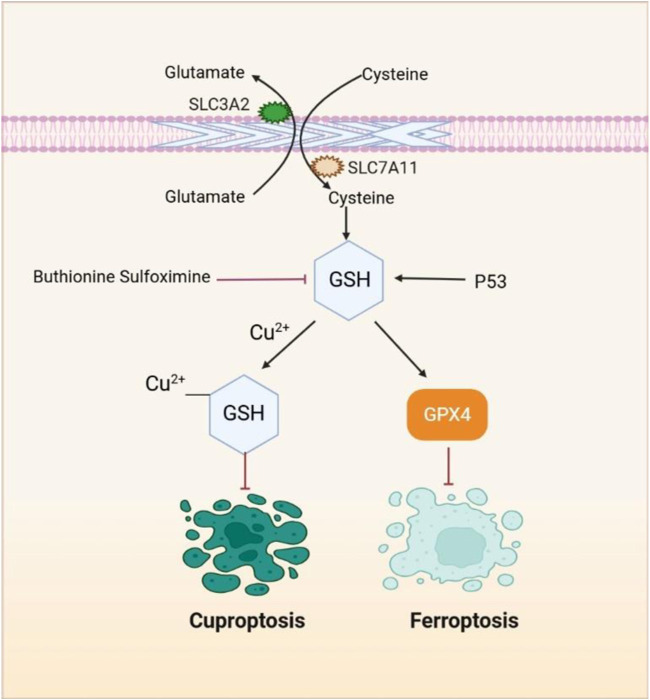
GSH, a pivotal element, plays a crucial role in both the ferroptosis and cuproptosis pathways. SLC7A11 facilitates the transport of cystine into cells, which is then used for the synthesis of GSH. As a potent reducing agent, GSH serves as a cofactor for GPX4, enabling GPX4 to degrade lipid peroxides and suppress lipid peroxidation and ferroptosis. Additionally, GSH functions as a copper chaperone, binding to copper and reducing its intracellular accumulation, thus preventing cuproptosis. Compounds such as buthionine sulfoximine induce both ferroptosis and cuproptosis by inhibiting GSH synthesis.

Depending on the cellular context, p53, a tumor suppressor, can inhibit or promote ferroptosis. The p53/p21 pathway activation enhances GSH synthesis, inhibiting phospholipid peroxidation and preventing ferroptosis (Tang D. et al., 2020). It is noteworthy that when lipid peroxidation damage is mild and repairable, p53 acts to prevent ferroptosis by promoting cellular repair mechanisms. However, when the damage is extensive or irreparable, p53 triggers ferroptosis to eliminate the damaged cells (Xu R. et al., 2023). The canonical p53 pathway involved in ferroptosis regulation includes modulation of iron metabolism. The p53 enhances the entry of Fe^2^⁺ into cells by upregulating the expression of TFR1 through lncRNA PVT1, and it also stimulates the production of reactive iron species by regulating *SLC25A28* (Zhang Z. et al., 2020) and *FDXR* (Zhang et al., 2017), thereby facilitating ferroptosis (Xu R. et al., 2023). Moreover, buthionine sulfoximine, a GSH synthesis inhibitor, induces both ferroptosis and cuproptosis, making it a promising therapeutic candidate (Figure 4) (Jiang et al., 2015; Li Y. et al., 2020; Luo et al., 2021; Rappa et al., 2003; Tang D. et al., 2021; Tsvetkov et al., 2022). However, further research and clinical trials are needed to explore its potential.

#### 2.3.1 GSH metabolism and ferroptosis

In breast cancer, elevated GSH levels are frequently observed, especially in TNBC, potentially attributed to estrogen-mediated downregulation of transferrin receptor expression (Ge A. et al., 2024). Such heightened GSH levels contribute to resistance against ferroptosis and other cell death modalities, fostering cancer progression and therapeutic resistance (Liu Y. et al., 2022). Notably, GSH-rich environments in breast cancer cells counteract the cytotoxic effects of anticancer agents like cisplatin and paclitaxel by neutralizing ROS, thereby conferring treatment resistance. However, interventions targeting GSH metabolism, such as benzothiazole-mediated GSH inhibition, have demonstrated promising efficacy in sensitizing breast cancer cells to conventional treatments (Ge A. et al., 2024). Therefore, manipulating GSH levels holds therapeutic promise in breast cancer management. Strategies aimed at GSH depletion, achieved through inhibition of the cystine/glutamate antiporter or GPX4 inactivation, induce ferroptosis and enhance treatment efficacy (Ge A. et al., 2024; Xu C. et al., 2023). Reducing GSH levels has been shown to alleviate radiation resistance, particularly through hypoxia-inducible factor-1 (HIF-1) mediated metabolic reprogramming. Clinical implications include the development of therapeutic approaches targeting GSH metabolism, combining therapies integrating ferroptosis inducers with conventional treatments, and identifying GSH levels and ferroptosis-related gene expression as predictive biomarkers (Xu C. et al., 2023; Yang F. et al., 2023; Zhou T. J. et al., 2024). This comprehensive understanding of the interplay between GSH metabolism and ferroptosis offers novel avenues for refining breast cancer therapy and improving patient outcomes.

#### 2.3.2 GSH metabolism and cuproptosis

Ferroptosis inducers like sorafenib and erastin downregulate GSH synthesis, decreasing intracellular GSH levels. This refers to increased cuproptosis, compromising copper-chelating capacity and promoting labile copper accumulation. Restoring GSH levels reversed the sensitizing effect of ferroptosis inducers on copper-induced cytotoxicity and cuproptosis. Combining GSH synthesis inhibitors such as buthionine sulfoximine with copper ionophores could be a potential therapeutic approach for inducing cuproptosis in breast cancer cells. However, specific studies are needed to explore their efficacy and safety (Mao et al., 2024; Wang W. et al., 2023; Wang Y. et al., 2024).

### 2.4 Autophagy

**FIGURE 5:**
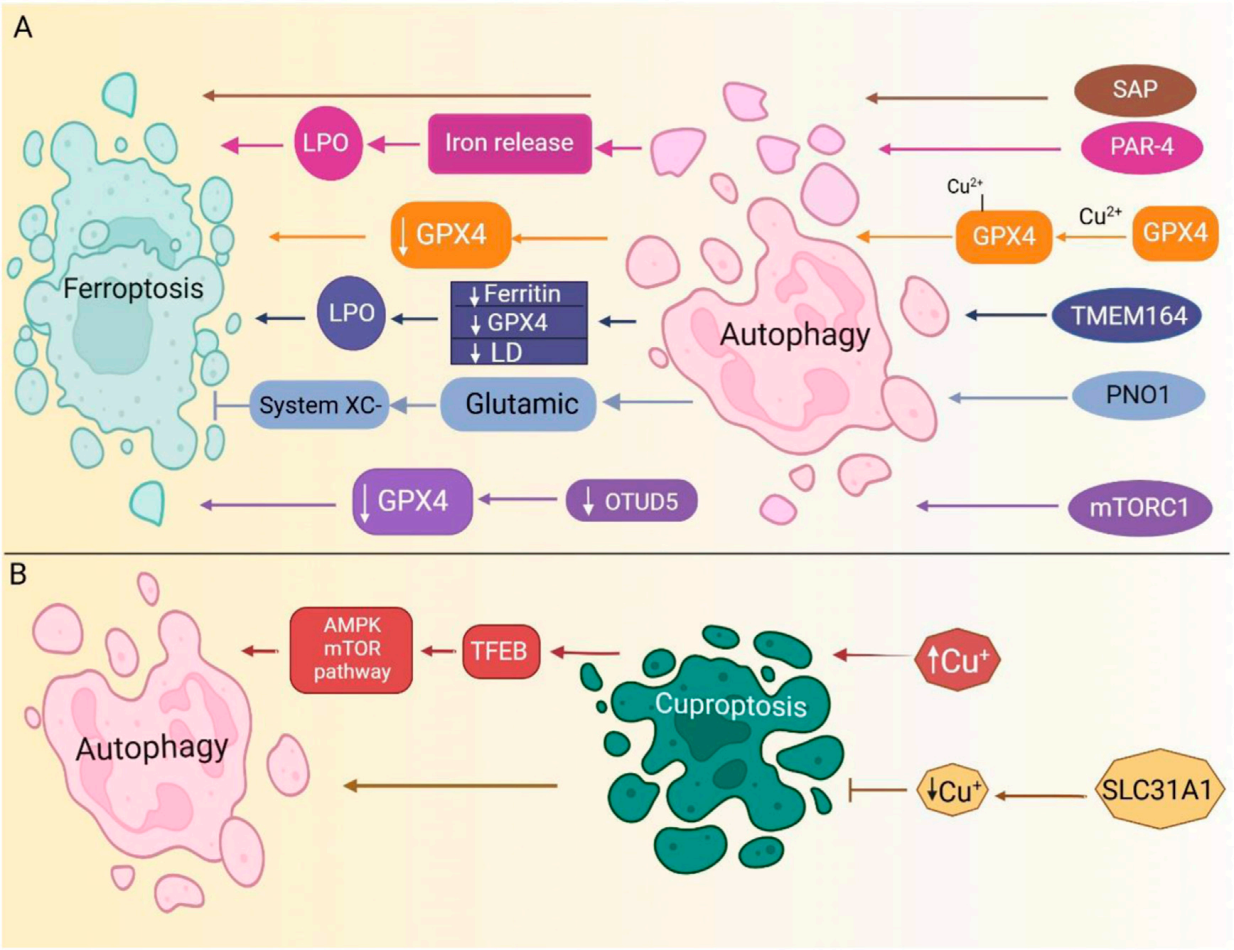
Autophagy serves as a key regulator of both ferroptosis and cuproptosis. **(A)**. Several signaling pathways induce autophagy. PAR-4 upregulation triggers ferritinophagy, resulting in free iron release, increased lipid peroxidation (LPO), and ferroptosis induction. Additionally, mTORC1-mediated autophagy downregulates OTUD5, leading to GPX4 reduction and subsequent ferroptosis. SAP upregulation further enhances autophagy, driving ferroptosis. **(B)**. Modulating copper uptake regulates autophagy pathways. For further details, please refer to the main text. (Routes are color-coded to indicate distinct pathways). SAP, severe acute pancreatitis; LPO, lipid peroxidation; PAR-4, Prostate apoptosis response-4; OTUD5, OTU deubiquitinase 5; mTORC1, mammalian target of rapamycin complex 1; LD, lipid droplets.

Autophagy is pivotal for maintaining cellular homeostasis under physiological and pathological conditions by facilitating the sequestration and degradation of various cellular components to meet metabolic demands and ensure organelle renewal (Levine and Kroemer, 2019; Mizushima and Komatsu, 2011). Perturbations in copper homeostasis are widely acknowledged to regulate not only ferroptosis and cuproptosis pathways but also the activation of autophagy (Li Y. et al., 2021; Xue et al., 2023a; Yang et al., 2021). Excess copper in cells activates transcription factors, upregulates *ATG5* expression, and modulates the AMPK-mTOR pathway, thereby inducing autophagy (Xue et al., 2023a; Yang et al., 2021) and establishing potential cross-talk between ferroptosis and cuproptosis (Figure 5).

#### 2.4.1 Autophagy and ferroptosis

The interaction between autophagy and ferroptosis in breast cancer is multifaceted, with autophagy exhibiting both promoting and protective roles in ferroptosis induction. Autophagy facilitates ferroptosis by promoting iron accumulation and lipid peroxidation, which are crucial events in ferroptosis initiation. Specific autophagic processes such as ferritinophagy and lipophagy release iron and lipids, promoting ferroptosis (Gao et al., 2016; Li J. et al., 2021; Liu et al., 2020). Conversely, inhibition of autophagy-related genes or cargo receptors impedes ferritin degradation, thus preventing ferroptosis onset. Moreover, ferroptosis inducers like erastin and sulfasalazine can induce autophagy in breast cancer cells. This induction of autophagy, however, seems to diminish the efficacy of ferroptosis inducers in killing breast cancer cells. Inhibiting autophagy in such conditions sensitizes breast cancer cells to ferroptosis inducers (Chipurupalli et al., 2023). The interplay among ferroptosis, autophagy, and iron metabolism underscores their interconnectedness in breast cancer pathophysiology.

Both ferroptosis and autophagy are driven by iron-dependent ROS generation, and alterations in intracellular iron levels and ferritin expression contribute to their respective cell death mechanisms. Heme oxygenase-1, which increases the cellular free iron pool, can further enhance ferroptosis in breast cancer cells (Chipurupalli et al., 2023; Lee et al., 2023; Ma et al., 2017; Xu C. et al., 2023). Moreover, in pancreatic cancer, transmembrane protein 164 (TMEM164) has been shown to activate autophagy, leading to the degradation of ferritin, GPX4, and lipid droplets. This process increases iron accumulation and lipid peroxidation, thereby promoting ferroptosis in cancer cells (Liu J. et al., 2023). Autophagy, therefore, plays a pivotal role in facilitating ferroptosis in cancer therapy. Interestingly, copper not only induces proteotoxic stress but also drives ferroptosis by directly binding to GPX4, resulting in the formation of GPX4 aggregates. These aggregates undergo autophagic degradation, further inducing ferroptosis (Xue et al., 2023b). Furthermore, in glioblastoma (GBM), inhibiting autophagy increases the sensitivity of GBM stem cells to temozolomide treatment by inducing ferroptosis (Buccarelli et al., 2018). Conversely, in hepatocellular carcinoma, autophagy driven by PNO1, a known ferroptosis inhibitor, enhances glutamate synthesis, activating system Xc− and ultimately preventing ferroptosis (Hu X. et al., 2022). In non-small cell lung cancer, curcumin induces ferroptosis through the activation of autophagy (Tang X. et al., 2021). These studies suggest that autophagy can either promote or suppress ferroptosis depending on the cancer type, which may be influenced by factors such as tumor heterogeneity or varying cellular environments. Further research is needed to clarify these interactions. Therapeutically, simultaneously targeting both ferroptosis and autophagy pathways has emerged as a promising strategy to overcome treatment resistance in breast cancer. Combination therapies incorporating ferroptosis inducers and autophagy modulators can potentially improve treatment effectiveness.

#### 2.4.2 Autophagy and cuproptosis

Disturbance of copper homeostasis modulates ferroptosis and cuproptosis and initiates autophagy pathways (Li Y. et al., 2021; Xue et al., 2023a; Yang et al., 2021). The intracellular accumulation of copper activates transcription factor EB (TFEB), upregulates *ATG5*, sequestosome 1 *(SQSTM1)*, and microtubule-associated protein 1 light chain 3 *(MAP1LC3)* expression, and modulates the AMPK-mTOR pathway, thereby stimulating autophagy (Xue et al., 2023a; Yang et al., 2021). Inhibition of the copper transporter SLC31A1 in pancreatic cancer cells results in decreased intracellular copper concentrations and enhances autophagy (Yu et al., 2019). However, the interplay between cuproptosis and autophagy in breast cancer remains ambiguous, yet it is postulated that disruptions in copper homeostasis may promote autophagy. Further investigation into this crosstalk is warranted to elucidate the underlying mechanisms and implications for cancer pathogenesis and treatment strategies.

## 3 The prognostic significance of genes associated with ferroptosis and cuproptosis in breast cancer

Identifying prognostic biomarkers/models to predict cancer progression is critical for two reasons. First, these biomarkers/models have practical clinical applications in patient treatment. Second, studying these biomarkers will provide new insights into disease mechanisms and the molecular processes driving pathological behavior (Passaro et al., 2024). Prognostic models integrating ferroptosis and cuproptosis genes show great potential for improving breast cancer prognosis and treatment (Li J. et al., 2023a). Breast cancer demonstrates extensive heterogeneity at both inter- and intra-tumoral levels, driven by distinct genomic, epigenomic, transcriptomic, and proteomic variations that contribute to differential therapeutic responses and disease progression. Ferroptosis is particularly relevant in TNBC due to its increased sensitivity to ferroptosis induction, though quantification across subtypes remains challenging. Similarly, cuproptosis may impact tumor cells with elevated copper levels. This heterogeneity complicates treatment, underscoring the need for biomarker-based studies to optimize targeted therapeutic approaches. The gene signatures effectively predict patient outcomes and overall survival. Additionally, they correlate with the tumor immune microenvironment and response to immunotherapy, guiding personalized treatment strategies like combining immunotherapy with ferroptosis/cuproptosis inducers for better outcomes (Li et al., 2023b; Li J. et al., 2022; Xu L. et al., 2023). Table 1 provides an overview of the impact of ferroptosis and cuproptosis genes on breast cancer prognosis.

**TABLE 1:** Prognostic impact of genes associated with ferroptosis and cuproptosis in breast cancer.

Type of cell death	Genes involved	Outcome	Value adding	Future necessity	Reference
Ferroptosis	*NDUFA13*	*NDUFA13* expression serves as a positive biomarker for the activation of the ferroptosis pathway in breast cancer patients.	Quantifying alterations in the programmed cell death pathways revealed a strong correlation between ferroptosis and early recurrence and progression in breast cancer (BRCA). Ferroptosis pathway status revealed intra-tumor heterogeneity, with mechanistic analyses highlighting distinct tumor microenvironments and immune functions across patients.	A detailed investigation into the role of *NDUFA13* in activating the ferroptosis pathway may offer crucial insights for developing targeted therapies.	Li Y. et al. (2022)
	*CISD1, CARS1, SLC7A11, ALOX15*	Overall survival prediction.	1. *CISD1*: A mitochondrial protein-coding gene that modulates ferroptosis through its influence on mitochondrial iron accumulation. It is linked to tumor growth and represents a promising therapeutic target. 2. *ALOX15*: Potential cancer treatment target; its expression predicts clinical outcomes for patients with lymph node metastasis and malignant tumors. 3. *CARS1*: Involved in cysteine metabolism and glutathione synthesis, predicting ferroptosis in kidney cancer and regulating cellular oxidative environment. 4. *SLC7A11*: Associated with amino acid transport, regulation of glutathione homeostasis, and defense against oxidative stress; crucial for oxidative stress signaling, cell proliferation, and tumor growth.	Future research could aim to incorporate additional clinical information that may enhance the comprehensiveness of the database utilized in this study.	Wang L. et al. (2022)
	*ALOX15, CHAC1, CISD1, CS, SLC7A11, EMC2, G6PD, ACSF2*	Overall survival prediction.	An eight-gene prediction model was developed and validated across TCGA-BRCA and METABRIC cohorts. It stratified patients into high- or low-risk groups, with the risk score serving as an independent predictor for overall survival. Immune cell and pathway analyses showed significant differences, highlighting the model’s clinical potential.	To enhance clinical applicability, future studies should incorporate real-world clinical data, including short-term treatment responses and detailed patient histories (e.g., chemotherapy, tumor stage, and metastatic lymph nodes). Additionally, further validation of the prognostic model in diverse patient cohorts beyond public databases is needed to strengthen its clinical relevance.	Li H. et al. (2021)
	*PROM2, ANO6, FLT3, G6PD, IFNG, NGB, PIK3CA, SLC1A4, TP63*	Overall survival prediction.	Five genes *(ANO6, FLT3, G6PD, IFNG, and PIK3CA)* were identified as drivers of ferroptosis progression, while two genes *(PROM2* and *TP63)* act as suppressors. Additionally, *NGB* and *SLC1A4* function as markers in the context of ferroptosis.	Future research should address the limitations of this study by incorporating prospective data, expanding the use of multiple markers to identify key prognostic genes, and exploring environmental and genetic factors related to BRCA. Additionally, further functional analyses on immunity and the ferroptosis-related risk score are essential.	Lu et al. (2022)
	*GPX4, SLC7A11, G6PD, EMC2, CISD1, ALOX15, ACSF2, GCLC, CS*	Overall survival prediction.	Genes pivotal in BRCA pathogenesis have been incorporated into a prognostic model. Researchers identified notable variances in immune-related cellular compositions and pathways between high- and low-risk groups. These findings offer promising insights into the prospective utilization of immunotherapy in BRCA management.	Future research demands the role of immunotherapy in treatment plans based on the immune cell levels and pathway differences in various BRCA risk groups.	Wang D. et al. (2021)
	*IFNG, ANO6, CHAC1, FLT3, JUN, MT3, PTGS2, SLC1A4, SLC7A5, TF, TP63*	Overall survival prediction.	Revealed ten immune targets were identified *CD80, CD276, BTN1A1, HHLA2, LILRA2, NCR3LG1, NECTIN3, PVR, SLAMF9, TNFSF4.* Most of these targets have not been assessed as potential targets for breast cancer treatment or studied for their connections to ferroptosis. This study unveiled the potential of these targets as therapeutic options related to ferroptosis in BRCA, introducing a new avenue in BRCA immunotherapy.	1. Explore lncRNA, miRNA, and other factors to broaden the analysis scope and improve prognostic models. 2. Further investigation: Needed for genetic targets associated with ferroptosis in breast cancer; clinical validation pending, requiring prospective trials for real-world confirmation.	Li Q. et al. (2023)
	Protective genes *(ALB, ANGPTL7, NGB, IL6)* and risk-related genes *(BLOC1S5-TXNDC5)*	Overall survival prediction.	The diagnostic signature accurately predicts BRCA risk levels, validated in an independent cohort and clinical tissue samples using PCR for mRNA detection and Western blot analysis for protein detection in BRCA cell lines.	Future research should explore how immune microenvironment invasion affects the prognosis of new genetic traits to discover novel diagnostic and treatment methods for BRCA.	Wang N. et al. (2022)
	*CHAC1, FANCD2, AIFM2, G6PD, HMOX1, CARS1*	Predicting Disease-Free Survival, Overall Survival prediction.	The constructed model is capable of categorizing patients by clinical and molecular characteristics.	Further clinical validation and investigation are needed.	Zhu J. et al. (2022)
	*BCL2, SUSD3, SERPINA3, AGBL2, SEC14L2, ELOVL2, FGD3, CASC1, TPRG1*	Overall survival prediction.	The study claimed that ferroptosis scores varied significantly based on patient age, TP53/PIK3CA mutations, PAM50 molecular subtypes, and clinical stages. Assessing ferroptosis activation in BRCA could enhance understanding of tumor microenvironment infiltration and inform more effective immunotherapy strategies.	Future research should aim to validate findings with larger sample sizes, confirm the accuracy of TCGA-BRCA data, and integrate additional factors such as methylation, copy number variation, and proteomic data for a more comprehensive analysis.	Yin and Tang (2022)
	*IFNG, FH, MT1G, CISD1, GABARAPL1, SLC1A5, SLC2A12, SLC2A8, WIPI1, NRAS, BRD4, FADS2, DUOX1, HSF1, TFAP2C*	Overall survival prediction.	A 15-gene ferroptosis-related prognostic model for TNBC was developed, demonstrating strong accuracy in predicting overall survival. This model could serve as a valuable tool for prognosis prediction and guide clinical practice for TNBC patients.	Future studies should focus on validating the predictive power of the prognostic model in external cohorts and further exploring the relationship between the immune microenvironment and the risk score.	Wu et al. (2022)
	*TFR2* *RGS4* *ZNF36*	Overall survival.	Pioneering study to demonstrate the regulatory influence of *TFR2* on ferroptosis *in vitro* within TNBC cells. Downregulation of *TFR2* suppresses the proliferation of TNBC cells and enhances ferroptosis in TNBC cell lines.	Further research is warranted to fully elucidate the role of *TFR2* in TNBC in subsequent studies.	Du, et al. (2024)
	*ACSL4, GPX4*	Higher *ACSL4*: better overall survival; Higher *GPX4*: improved distant metastasis-free survival. *ACSL4, GPX4*, and their combination are independent prognostic factors for disease-free survival.	The high *ACSL4*/low *GPX4* profile holds significant practical value in predicting pathological complete response to neoadjuvant chemotherapy in breast cancer. A strategy to enhance chemosensitivity through the induction of ferroptosis has been identified.	Fundamental research is essential to elucidate the mechanisms by which ferroptosis impacts chemosensitivity.	Li J. et al. (2021)
Cuproptosis	*PGK1, SLC52A2, SEC14L2, RAD23B, SLC16A6, CCL5, MAL2*	Predicting chemotherapy efficacy and overall survival.	*RAD23B* has been recognized as a potential favorable target associated with BRCA progression, drug resistance, and poor prognosis in BRCA patients. This conclusion was drawn using real-time RT-PCR, cell viability assays, and IC50 testing. The cuproptosis-related scoring system proved valuable for clinical patient stratification, predicting chemotherapy efficacy, and patient prognosis, while also providing a foundation for deeper investigation into cuproptosis’ role in BRCA progression.	Future research should include prospective studies and experimental validation both *in vivo* and *in vitro* to confirm the findings. Additionally, further exploration of the molecular mechanisms by which cuproptosis regulates BRCA progression is necessary.	Song et al. (2022)
	*FDX1, LIAS, LIPT1, DLD, DLAT, PDHA1, PDHB, GLS, MTF1, CDKN2A*	Overall survival prediction and immune response.	This study is the first to highlight the pivotal role of cuproptosis in influencing clinical outcomes and the diversity of the tumor microenvironment (TME) in BRCA. Evaluating cuproptosis patterns offers valuable insights for prognostic prediction, TME cell infiltration, and guides more effective chemotherapy and immunotherapy approaches.	Further studies are needed to explore the therapeutic implications of these findings in BRCA.	Li W. et al. (2022)
	*MTF1, DKN2A, PDHA1, DLD, LIPT1, FDX1*	Overall survival prediction and relapse-free survival.	Successfully incorporated a six-gene signature model for BRCA patients. Moreover, the research identified the previously unstudied lncRNA XIST/miR-92b-3p/MTF1 regulatory axis in breast cancer.	Future research should focus on validating the cuproptosis-related gene signature in diverse cohorts and exploring the functional roles of the lncRNA XIST/miR-92b-3p/MTF1 axis in breast cancer progression.	Jiang B. et al. (2022)
	*SLC7A5, STC2, MAPT, TFF1, CHAD, GREB1, SCUBE2, SUSD3, MMP7, CHI3L1, FABP7*	Overall survival prediction.	The study highlighted the role of cuproptosis-related genes in the occurrence, progression, and prognosis of BRCA, offering a promising signature for predicting drug response and guiding immunotherapy and chemotherapy strategies. This work provides novel insights into therapeutic approaches for breast cancer prevention and treatment.	Future studies should focus on validating the prognostic model with biological evidence and exploring single-cell RNA-seq to better identify specific cell populations involved in breast cancer prognosis, enhancing the accuracy of the gene expression-based signatures.	Zheng et al. (2023)
	*PGK1, MRPL39, COPB2, HSPH1, NFKBIA, PRDX1, PCMT1, MPZL3, DLG3, DIP2B, LACTB2*	Overall survival, immune response, and drug sensitivity predictor.	The constructed risk signature demonstrated high prognostic accuracy and effectively predicted BRCA molecular subtypes. It showed significant correlations with PR, ER, and HER-2 expression levels, which are critical factors influencing the survival of breast cancer patients. Differential PD-L1 expression, negatively correlated with the risk score, was verified, indicating the potential of this risk signature to guide chemotherapy drug treatment.	Future functional studies are needed to understand the mechanisms through which cuproptosis-associated genes influence breast cancer progression.	Shen L. et al. (2022)
	*SLC31A1*	Potential predictor for diagnosis, prognosis, and therapeutic response of breast cancer.	*SLC31A1* emerged as a prospective cuproptosis-related gene in breast cancer, displaying significant upregulation. It exhibited promising potential in forecasting prognosis, aiding in diagnosis, and predicting drug sensitivity in breast cancer cases.	Aggregation of more cuproptosis genes is expected to incorporate a better prognostic model for breast cancer patients.	Li L. et al. (2022)
	*NLRP3, LIPT1, PDHA1 DLST*	Overall survival prediction.	This research introduces a novel gene signature for predicting breast cancer prognosis, providing a foundation for future studies on the relationship between CRGs and the tumor immune microenvironment. NK cell activation as potential promising targets for tumor immunotherapy.	In forthcoming investigations, the aim should be to integrate cuproptosis-related genes with other pivotal molecular and clinically distinct prognostic elements to develop advanced risk models, enhancing patient stratification.	Zhou et al. (2022)
	*PDHA1*	Overall survival prediction and immune response.	The study uncovers that *PDHA1* could act as an autonomous prognostic biomarker and a promising target for immunotherapeutic approaches in breast cancer.	*PDHA1* is implicated in regulating the biological processes and clinical outcomes of breast cancer. However, further confirmation through rigorous and comprehensive molecular experiments is warranted.	Huang et al. (2022)
	*ATOX1, DLAT, SLC31A2, SLC25A3*	Survival prediction.	This study established a prognostic model based on a four-CRG signature, identifying *DLAT* as an independent prognostic factor associated with resistance to HER-2-targeted therapy in HER-2-positive breast cancer patients.	Future research should explore other potential CRGs related to cuproptosis, investigate the functional roles of the four CRGs, especially *DLAT*, in HER-2-positive BRCA, and examine *DLAT’s* impact on anti-tumor immunity in these patients.	Sha R. et al. (2024)
	*LIAS, FDX1, LIPT1, DLD, PDH1, ATP7B, CDKN2A*	Invasive disease-free survival (iDFS) or relapse-free survival RFS.	The developed prognostic nomogram model exhibited predictive solid capability for the 7-year relapse-free survival RFS of ER+ EBC patients. When combined with other clinical features, the CRG score could serve as a practical predictor for long-term outcomes in patients with ER+ EBC.	Future research should extend follow-up periods for ER + BRCA patients, conduct subgroup analyses, expand sample sizes, incorporate clinical features, and consider overall survival as an endpoint for comprehensive patient outcomes.	Fan Y. et al. (2023)
	*NFE2L2, NLRP3, ATP7A, ATP7B, SLC31A1, LIAS, LIPT1, GLS, DLAT, PDHB, DLST*	Predicts survival and immune response.	The signature may influence the progression of TNBC, modulate the tumor immune microenvironment, and affect patient survival. Crucial CRGs in TNBC interact and might be affected by mutations related to BRCA. An 11-gene risk model forecasts survival throughout 5–15 years.	Biological mechanisms should be elucidated.	Shi et al. (2023)
	*PTPRN2, SCARB1, SLC37A2, YES1, LY6D, NOTCH3*	Overall survival prediction.	This study highlighted the role of cuproptosis in TNBC development and its interaction with the tumor immune microenvironment. Identified three cuproptosis-related molecular subtypes, aiding personalized immunotherapy, and developed a robust six-gene risk model for TNBC prognosis prediction.	Future research should validate the cuproptosis-related subtypes and risk model in experimental settings and prospective studies while exploring their role in guiding immunotherapy for TNBC.	Zhu B. et al. (2022)
	*FDX1, LIAS, LIPT1, DLD, DLAT, PDHA1, PDHB, MTF1, GLS, CDKN2A*	Cuproptosis role in determining the clinical outcome and shaping the diversity and complexity of the TME.	The cuproptosis score helps evaluate cuproptosis patterns, prognosis, and TME cell infiltration, guiding treatment decisions. It predicts responses to hormone therapy, chemotherapy, and anti-PD-L1/PD1 immunotherapy. Targeting CRGs or cuproptosis-related genes could modify TME characteristics, enhancing immunotherapy efficacy and enabling personalized BRCA treatments.	Future research should validate the scoring system with large-scale, multi-center data and investigate the mechanisms between CRGs, TME infiltration, and immunotherapy efficacy. Continuous monitoring of CRGs and immune markers is essential for optimizing treatment strategies.	Li W. et al. (2022)
Ferroptosis/Cuproptosis Association	*ANKRD52, HOXC10, KNOP1, SGPP1, TRIM45*	Potential predictor for survival, patient immunotherapy, and immune cell infiltration.	The predictive model is tailored for BRCA patients, innovatively linking ferroptosis and cuproptosis. It accurately forecasts prognosis, evaluates immune infiltration, identifies resistance patterns, and offers guidance to clinicians for personalized treatment strategies.	Further research should investigate the linkage of ferroptosis and cuproptosis genes to predict overall survival in BRCA patients.	Li et al. (2023b)

*Note: BRCA*, Breast cancer; *ER,* Estrogen receptor; *PR,* Progesterone receptor; *FRGS,* Ferroptosis-related genes; *CRGS,* Cuproptosis-related genes; *TNBC,* Triple-negative breast cancer; *lncRNA,* Long noncoding RNA; *miRNA,* microRNA; *PCR,* polymerase chain reaction; *mRNA,* Messenger RNA; *IHC,* Immunohistochemistry; *iDFS,* Invasive disease-free survival; *DFS,* Disease-free survival; *TME,* Tumor microenvironment; *RFS*, relapse-free survival.

### 3.1 Ferroptosis-associated genes and the prognosis of breast cancer

Ferroptosis has emerged as a promising therapeutic avenue for treating breast cancer (Li Z. et al., 2020). Notably, research indicates that ferroptosis-related marker genes have the potential to serve as novel biomarkers for predicting the prognosis of breast cancer patients (Yang et al., 2021; Zhou L. et al., 2023). In particular, ferroptosis pathway status was significantly associated with clinical outcomes and intra-tumoral heterogeneity in breast cancer patients, as *NDUFA13* expression was identified as a positive biomarker for activating the ferroptosis pathway in breast cancer patients (Li Y. et al., 2022). Thus, an in-depth exploration of the involvement of ferroptosis-related genes underscores their significance in determining breast cancer prognosis, overall survival, and guiding treatment approaches. Building on this foundation, a recent study introduced a novel prognostic model for breast cancer, that integrates four ferroptosis-related genes *(CISD1, ALOX15, CARS1, and SLC7A11)* (Wang L. et al., 2022). Furthermore, the model independently and accurately predicted overall survival in breast cancer patients. Significantly, a nomogram was created to provide precise prognostic predictions for breast cancer individuals (Wang L. et al., 2022). In a related study, researchers constructed an eight-gene model associated with ferroptosis to predict breast cancer patient’s prognosis. This model effectively categorized patients into high- or low-risk groups. The inclusion of these 8 genes *(ALOX15, CHAC1, CISD1, CS, SLC7A11, EMC2, G6PD, and ACSF2)* is highly valuable for prognostic prediction in breast cancer patients (Li H. et al., 2021).

Moreover, another novel prognostic model, composed of nine ferroptosis-related genes has demonstrated significant accuracy in predicting survival outcomes in breast cancer patients. These genes included *PROM2, ANO6, FLT3, G6PD, IFNG, NGB, PIK3CA, SLC1A4,* and *TP63*. Among them, five genes *(ANO6, FLT3, G6PD, IFNG, and PIK3CA)* were identified as drivers contributing to ferroptosis progression. Conversely, two genes *(PROM2 and TP63)* exhibit suppressor functions, while the remaining two genes *(NGB and SLC1A4)* serve as markers within the context of ferroptosis (Lu et al., 2022). Additionally, a model comprising nine ferroptosis-related genes *(ALOX15, CISD1, CS, GCLC, GPX4, SLC7A11, EMC2, G6PD,* and *ACSF2)* was employed in this study. It is believed that it holds promise as an innovative biomarker for predicting the prognosis of breast cancer patients (Wang D. et al., 2021). Expanding this line of inquiry, a survival prediction model was developed using eleven prognostic-related genes *(TP63, IFNG, MT3, ANO6, FLT3, PTGS2, SLC1A4, JUN, SLC7A5, CHAC1,* and *TF)* derived from differentially expressed genes (DEGs). The model exhibited strong predictive capacity in breast cancer patients (Li Q. et al., 2023).

In addition, a study developed a gene signature focusing on genetic diversity, consisting of protective genes *(ALB, ANGPTL7, NGB,* and *IL6)* and risk-related genes *(BLOC1S5-TXNDC5)*. This diagnostic signature accurately predicts breast cancer risk levels. Researchers have validated its reliability and applicability through independent cohort analysis, PCR-based mRNA detection in clinical tissue samples, and Western blot analysis in BRCA cell lines (Wang N. et al., 2022). Another study established a ferroptosis activation-risk-related score model (FeAS) that included 13 genes, which were verified using machine learning and single-cell RNA sequencing data. The model showed promising prognostic capacity and could guide clinical treatment to prevent drug resistance, influencing breast cancer patient outcomes (Liu S. et al., 2023). A novel prediction signature comprising six genes *(CARS1, CHAC1, FANCD2, AIFM2, G6PD,* and *HMOX1)* was developed utilizing the least absolute shrinkage and selection operator (LASSO) Cox regression methodology. The expression levels of these six genes were subsequently validated through real-time quantitative polymerase chain reaction and immunohistochemistry assays using samples from the Human Protein Atlas. Notably, patients categorized into the high-risk group based on this signature demonstrated a greater propensity for relapse or metastasis. Furthermore, the risk score derived from this signature emerged as an independent prognostic factor for disease-free survival (Zhu J. et al., 2022). A study identified nine ferroptosis-related genes with prognostic value in breast cancer: *BCL2, SUSD3, SERPINA3, AGBL2, SEC14L2, ELOVL2, FGD3, CASC1,* and *TPRG1*. The prognostic model based on these genes showed that patients with high ferroptosis scores had significantly better overall survival (OS) than those with low ferroptosis scores. Additionally, the models demonstrated high reliability in predicting one-, three-, and five-year survival rates through time-dependent ROC curve analysis (Yin and Tang, 2022).

Continuing this trend, a prognostic prediction model consisting of 15 ferroptosis-related genes *(IFNG, FH, MT1G, CISD1, GABARAPL1, SLC1A5, SLC2A12, SLC2A8, WIPI1, NRAS, BRD4, FADS2, DUOX1, HSF1, and TFAP2C)* was established. The prognostic model exhibited high accuracy according to time-dependent ROC curve analysis, with AUCs of 0.948, 0.956, and 0.940 for the 1-, 3-, and 5-year intervals, respectively (Wu et al., 2022). The prognostic model offers a theoretical framework for precise prognosis prediction in clinical settings for triple-negative breast cancer (Wu et al., 2022). Significantly, another study identified MTHFD2 as a significant molecular biomarker for prognostic prediction and a novel therapeutic target in TNBC. Additionally, MTHFD2 was identified as a potential regulatory gene for ferroptosis in TNBC. *In vitro* experiments revealed that MTHFD2 knockdown inhibited proliferation, induced apoptosis, and suppressed migration and invasion in TNBC cells (Zhang H. et al., 2023). Furthermore, a novel predictive signature comprising three ferroptosis-related genes, namely *TFR2, ZNP36,* and *RGS4*, was developed to predict prognostic outcomes in TNBC cell lines (Yang et al., 2024a). Notably, the study documents the negative regulatory function of *TFR2* in TNBC ferroptosis, with *TFR2* downregulation leading to inhibited proliferation and ferroptosis induction in TNBC (Yang et al., 2024a). Lastly, a breakthrough documented for the first time that short-term treatment with endocrine agents can sensitize ER+ breast cancer cells to ferroptosis-inducing agents, suggesting a sensitization mechanism independent of genetic mutations. Building on this concept, researchers developed a 55-gene signature, referred to as the FERscore (Hu et al., 2024), specifically designed to predict the susceptibility of breast cancer to ferroptosis. Patients with breast cancer who had lower FERscores were associated with significantly improved survival outcomes. Data from both cell lines and primary tumor samples revealed that ER+ breast cancer typically exhibited lower FERscores compared to other subtypes. However, in endocrine-resistant ER+ tumor cells and residual tumors following endocrine therapy, the FERscore was markedly elevated. In breast cancer, higher FERscore levels were positively associated with traits such as mesenchymal phenotype, stemness, immune cell infiltration, and cancer-associated fibroblast (CAF) enrichment, while they were negatively associated with features like estrogen response and DNA repair capacity (Hu et al., 2024).

### 3.2 Cuproptosis-associated genes and prognosis of breast cancer

Cuproptosis can be regulated by specific genes known as cuproptosis-related regulators (CRRs), which include *DLD, PDHB, ATP7B, ATP7A, DLAT, DLST, SLC31A1, DBT, FDX1, LIPT1, LIAS, GCSH,* and *PDHA1* (Tsvetkov et al., 2022). Expanding on this, CRRs could enhance our understanding of cuproptosis in diseases, including breast cancer. Growing evidence indicates that cell death pattern signatures significantly predict prognosis, the tumor immune microenvironment (TIME), and immunotherapy response in breast cancer patients (Li Z. et al., 2022). Recently, signatures related to ferroptosis (Zhu L. et al., 2021), pyroptosis (Xu L et al., 2022), and necroptosis have been identified. The role of cuproptosis in breast cancer remains underexplored, necessitating further investigation into genetic changes in CRGs to uncover therapeutic opportunities. In line with this, a novel breast cancer signature consisting of CRGs such as PGK1, SLC52A2, and RAD23B has shown potential for prognosis prediction, with RAD23B emerging as a promising target linked to disease progression and drug resistance (Song et al., 2022).

Building on these findings, ten genes responsible for copper-induced cell death through genome-wide CRISPR-Cas9 loss-of-function screens and individual gene knockout studies. Seven of these genes *(FDX1, LIAS, LIPT1, DLD, DLAT, PDHA1,* and *PDHB)* promote cuproptosis, while the other three *(MTF1, GLS,* and *CDKN2A)* inhibit cuproptosis. Patients with luminal A and basal subtypes were classified into cluster1 and cluster2, respectively. The basal subtype is significantly associated with the worst prognosis in patients with breast cancer, whereas the luminal A subtype is linked to the best clinical outcomes. Furthermore, the cluster expression patterns of cuproptosis-related genes (CRGs) differed. Cluster1 exhibited increased expression of most cuproptosis-promoting genes *(LIPT1, LIAS, PDHB, FDX1, DLAT,* and *DLD)*, while cluster2 exhibited increased expression of cuproptosis-inhibiting genes *(CDKN2A* and *GLS)* and one cuproptosis-promoting gene *(PDHA1)*. This suggests that cuproptosis may inhibit breast cancer progression by inducing tumor cell death (Li W. et al., 2022). Additional studies have developed a cuproptosis-related signature with six genes *(MTF1, DKN2A, PDHA1, DLD, LIPT1, FDX1)* for breast cancer, which accurately predicted the OS rate (Jiang B et al., 2022). Another study underscored the ability of the *SLC7A5, STC2, MAPT, TFF1, CHAD, GREB1, SCUBE2, SUSD3, MMP7, CHI3L1,* and *FABP7* genes to predict the overall survival rate of breast cancer patients (Zheng et al., 2023). In parallel, a novel prognostic risk signature for breast cancer patients was constructed using 11 cuproptosis hub genes namely, *PGK1, MRPL39, COPB2, HSPH1, NFKBIA, PRDX1, PCMT1, MPZL3, DLG3, DIP2B, LACTB2* (Shen L. et al., 2022).

Recently, high *SLC31A1* expression in breast cancer patients has led to poor overall survival, distant metastasis-free survival, and relapse-free survival (RFS), suggesting that *SLC31A1* may be an unfavorable prognostic biomarker (Li X. et al., 2022). Moreover, elevated *SLC31A1* expression in breast cancer samples indicates poor prognosis, shorter overall survival, and a dysregulated immune response. Low levels predict sensitivity to CTLA4 inhibitors but inadequate response to paclitaxel (Li L. et al., 2022). Researchers developed a nomogram model utilizing cuproptosis-related genes *(NLRP3, LIPT1, PDHA1,* and *DLST)* and discovered that the signature derived from these genes effectively stratifies patient subtypes and correlates closely with the TME. Additionally, these genes were identified as independent prognostic indicators for breast cancer patients (Zhou et al., 2022). In addition, elevated *PDHA1* expression correlated with poorer outcomes in breast cancer patients. Furthermore, immune infiltration analysis of CRGs revealed that *PDHA1* expression is significantly associated with the infiltration levels of CD4^+^ memory T cells, M0 and M1 macrophages, and mast cells in breast cancer.

*PDHA1* has been reported to be an independent prognostic biomarker and a potential target for breast cancer immunotherapy (Huang et al., 2022). A study identified a novel prognostic model comprising four CRGs *(ATOX1, DLAT, SLC31A2,* and *SLC25A3)* for HER-2-positive breast cancer patients. Among HER-2-positive breast cancer patients, *DLAT* was confirmed to be downregulated and correlated with improved survival. Elevated *DLAT* expression was associated with resistance to HER-2-targeted therapy and sensitivity to immunotherapy (Sha R. et al., 2024). In this case-control study, high expression of *LIAS, LIPT1*, and *ATP7B*, along with low *CDKN2A* expression, was linked to improved invasive disease-free survival (iDFS). In the cohort study, high expression of *LIAS, FDX1, LIPT1, DLD, PDH1, and ATP7B*, coupled with low *CDKN2A* expression, was associated with favorable RFS in patients with estrogen receptor-positive early breast cancer (ER+ EBC). The developed prognostic nomogram model exhibited predictive solid capability for the 7-year RFS of ER+ EBC patients (Fan Y. et al., 2023). In addition, researchers developed an 11-gene risk model for TNBC treatment, targeting 11 key CRGs *(NFE2L2, NLRP3, ATP7A, ATP7B, SLC31A1, LIAS, LIPT1, GLS, DLAT, PDHB, DLST).* The model predicts 5–15-year survival with an AUC of 0.836 (Shi et al., 2023). Another six-gene risk model, including *PTPRN2, SCARB1, SLC37A2, YES1, LY6D,* and *NOTCH3,* has been proven effective and reliable in predicting the prognosis of triple-negative breast cancer patients. The accuracy of the risk model in predicting TNBC prognosis was enhanced by establishing a nomogram that outperformed the TNM staging system (Zhu B. et al., 2022). The findings revealed that CRGs may impact tumor immunity in TNBC, clinical features, and prognosis, making them valuable tools for patient prognosis prediction (Sha et al., 2022; Zhu B. et al., 2022).

## 4 The landscape of ferroptosis/cuproptosis-related lncRNA in the prognosis of breast cancer

Although approximately 75% of the genome is transcribed into RNA, only 3% is translated into mRNA, which encodes proteins. Most of the transcriptome consists of noncoding RNAs (ncRNAs) lacking protein-coding potential. These ncRNAs may be classified based on their length, structure, and origin, with the four major types relevant to breast cancer being microRNAs (miRNAs), circular RNAs (circRNAs), long non-coding RNAs (lncRNAs), and tRNA-derived small RNAs (tsRNAs). Each ncRNA type plays a distinct role in gene regulation and cancer biology.

Small non-coding RNAs (sncRNAs), including miRNAs, circRNAs, and tsRNAs, are generally shorter than 200 nucleotides. Despite their small size and comprising less than 1% of the human transcriptome, sncRNAs are critical regulators of gene expression and various cellular processes (Klimenko, 2017; Smal et al., 2024). MiRNAs function primarily by binding to target mRNAs, leading to their degradation or translational repression. CircRNAs, on the other hand, could act as miRNA sponges, modulating gene expression indirectly by sequestering miRNAs. TsRNAs, derived from tRNAs, have emerging roles in controlling translation and responding to cellular stress. The biogenesis of sncRNAs involves highly regulated processing pathways, such as the enzymatic cleavage of precursor miRNAs by Drosha and Dicer (Pagès et al., 2018; Russell et al., 2024).

In contrast, long non-coding RNAs (lncRNAs), which are over 200 nucleotides in length, are involved in a wider array of regulatory functions. These include chromatin remodeling, transcriptional regulation, and serving as molecular scaffolds (Li D. et al., 2022; Mattick et al., 2023). LncRNAs associated with ferroptosis and cuproptosis in breast cancer have shown promise as biomarkers for early detection, prognostic modeling, and therapeutic targeting. While miRNAs, circRNAs, and tsRNAs are also crucial in cancer biology, this manuscript primarily focuses on lncRNAs due to their significant involvement in regulating ferroptosis and cuproptosis pathways in breast cancer. Table 2 provides a detailed overview of how lncRNAs related to these processes influence breast cancer prognosis.

**TABLE 2:** Prognostic impact of lncRNAs linked with ferroptosis and cuproptosis in breast cancer.

Type of cell death	lncRNAs	No. of lncRNAs	Value adding	Limitations	Reference
Ferroptosis	KLHDC7B-DT, AC012213.3, LIPE-AS1, SIDT1-AS1, AC009171.2, AC137630.3, HSD11B1-AS1, LINC02446, TFAP2A-AS1, AC079298.3, YTHDF3-AS1	11	The identified ferroptosis-related lncRNAs show potential for assessing breast cancer risk and optimizing immunotherapy strategies. These lncRNAs can serve as prognostic markers, aiding in early detection, therapeutic targeting, and exploring the anti-tumor immune microenvironment, while also guiding clinical treatment approaches.	The specific mechanisms linking ferroptosis-related lncRNAs to anti-tumor immunity are still unclear, requiring more detailed research. Additionally, the retrospective nature of this study limits the findings, and the absence of data for each BRCA subtype prevents analysis of immune microenvironment characteristics. Experimental studies and future clinical trials are required to validate the model’s accuracy and practical application.	Jia et al. (2021)
	AL035661.1, ADAMTS9-AS1, AC078883.1, FTX, AC007686.3, CBR3-AS1, TMEM105	7	Bioinformatics analyses revealed seven ferroptosis- and immune-related differentially expressed lncRNAs (FI-DELs) that are significantly linked to overall survival in patients with breast infiltrating ductal and lobular carcinoma, offering potential prognostic and therapeutic insights.	Additional studies are required to confirm the clinical relevance and applicability of these seven biomarkers.	Wei et al. (2022)
	AL133467.1, LINC01235, AC072039.2, USP30-AS1, AC108474.1, MAPT-AS1, TDRKH-AS1, LIPE-AS1	8	LncRNA USP30-AS1, co-expressed with 9 ferroptosis-related genes (including 4 drivers, 2 suppressors, and 3 markers), was associated with longer overall survival in BRCA. LncRNA LIPE-AS1, co-expressed with 5 ferroptosis-related genes (4 drivers and 1 suppressor), was identified as a protective factor for prognosis. Similarly, AC108474.1, co-expressed with 5 genes (1 driver, 2 suppressors, and 2 markers), also showed protective prognostic value. These findings highlight their potential role in ferroptosis regulation and BRCA prognosis.	The study’s limitations include reliance on the TCGA database, requiring validation with prospective, multicenter, real-world data. Future research should experimentally explore the mechanisms linking ferroptosis-related lncRNAs with anti-tumor immunity and assess the ability of ferroptosis-inducing drugs to convert “cold” tumors into “hot” tumors.	Zhang K. et al. (2021)
	LINC01871, LINC00393, AC121247.2, LINC02384, LIPE-AS1, HSD11B1-AS1, AC010655.2, LINC01419, PTPRD-AS1, AC099329.2, OTUD6B-AS1, LINC02266	12	The study thoroughly examined the relationship between ferroptosis, the immune microenvironment, and BRCA prognosis, offering key insights to guide the development of combined immunotherapy strategies in clinical practice.	This study has limitations: reliance on TCGA data without external validation or real-world data, absence of experimental validation for target FRLncRNAs and immune mechanisms, and limited clinical information, such as missing factors in the risk score analysis. Future research should address these gaps with multi-database validation, experimental confirmation, and inclusion of more clinical parameters.	Xu Z. et al. (2021)
	LINC01152, AC004585.1, MAPT-IT1, AC026401.3	4	The identification of four ferroptosis-related differentially expressed lncRNAs (FR-DELs) offers potential biomarkers for the prognosis and diagnosis of BRCA, providing new insights into patient outcome prediction.	Despite several limitations, particularly the lack of clinical validation, future research should focus on validating the findings with real-world data and experimental studies to strengthen the clinical applicability.	Yao et al. (2022)
	CYTOR, LMNTD2-AS1, LYPLAL1-AS1, USP30-AS1, RHPN1, LINC01655, AP005131.2, AC004988.1, AC079289.3		Among identified lncRNAs, this study uncovers that CYTOR and USP30-AS1, are significantly upregulated in BRCA cell lines, potentially contributing to tumorigenesis. The developed prediction model highlights promising immune biomarkers for BRCA treatment and provides valuable insights into the ferroptosis-related lncRNA mechanisms in BRCA. The predictive model demonstrates strong potential as a sensitive marker for forecasting BRCA patient response to immunotherapy.	The absence of external validation and molecular subtype stratification may introduce bias. Future studies will incorporate multicenter data, validate the model with clinical samples, and further explore lncRNA interactions with ferroptosis.	Shen S. et al. (2022)
	LINC01235, LINC02166, AL133467.1, TGFB2-AS1, LINC02266	5	A 5 ferroptosis-related lncRNA signature was identified for predicting RFS in BRCA patients, offering potential biomarkers for clinical management, treatment response prediction, and therapeutic targets.	Future research will address missing clinical data from TCGA, conduct independent validation, and perform *in vitro and in vivo* experiments to clarify the regulatory mechanisms of ferroptosis-related lncRNAs in BRCA while refining the clinical application of the signature.	Wang Y. et al. (2022)
	PTPRG-AS1	1	The study identified that PTPRG-AS1, regulated by POU2F2, modulates ferroptosis and proliferation in TNBC via the miR-376c-3p/SLC7A11 axis, highlighting its potential as a therapeutic target for TNBC treatment. The POU2F2/PTPRG-AS1/miR-376c-3p/SLC7A11 axis holds potential as novel biomarkers and therapeutic targets for ferroptosis-mediated cancer therapy in TNBC.	Further *in vitro and in vivo* validation of the PTPRG-AS1/miR-376c-3p/SLC7A11 axis in larger cohorts, along with clinical studies, is needed to confirm its role in ferroptosis modulation and to explore its potential in therapeutic applications for TNBC.	Li J. et al. (2024)
Cuproptosis	GORAB-AS1, AC079922.2, AL589765.4, AC005696.4, CYTOR, ZNF197-AS1, AC002398.1, AL451085.3, YTHDF3-AS1, AC008771.1, LINC02446	11	A cuproptosis-related lncRNA signature was successfully constructed, providing an independent prognostic predictor for BRCA patients and offering insights into OS and clinical treatment outcomes, with potential for guiding personalized therapy.	Further investigation into the underlying mechanisms of cuproptosis-related lncRNAs in BRCA, along with validation in diverse cohorts, is essential to refine therapeutic targets and develop new biomarkers for improved clinical management.	Jiang Z. R. et al. (2022)
	USP2-AS1, NIFK-AS1	2	This study introduces a cuproptosis-related 2-lncRNA signature (BCCuS) that serves as an independent prognostic marker for BRCA, offering predictive value for overall survival, immune function, gene mutations, and treatment response. The signature also identifies potential therapeutic targets and drugs for personalized treatment strategies.	Further validation of BCCuS in larger, multi-center cohorts is essential, along with experimental studies to elucidate the role of these lncRNAs in cuproptosis-related mechanisms and their interaction with key cancer pathways.	Xu Q. T. et al. (2022)
	AL118556.1, AL451123.1, MFF-DT, AL133243.2, ZKSCAN7-AS1, AC012676.3, AC009506.1, AC079766.1, MIR1915HG, AC138028.2	10	The study establishes a novel prognostic model based on 10 cuproptosis-related lncRNAs, offering a reliable tool for predicting overall survival in BRCA patients. The model demonstrates superior diagnostic efficiency compared to other clinical features and highlights the role of tumor mutational burden (TMB) in survival outcomes, potentially guiding personalized therapeutic strategies.	Further validation using larger, independent cohorts and clinical trials is necessary to confirm the utility of the cuproptosis-related lncRNA model. Additionally, experimental studies are needed to elucidate the underlying mechanisms of these lncRNAs in cuproptosis regulation and their impact on immunotherapy efficacy.	Pan et al. (2023)
	ARHGAP28-AS1, LINC01711, LRRC8C-DT, PCAT18, SIAH2-AS1, TDRKH-AS1, SAMMSON, WDFY3-AS2, LINC00393	9	This study is the first to investigate the relationship between cuproptosis-related lncRNAs, the tumor immune microenvironment (TIME), and prognosis in BRCA. It highlights the potential of cuproptosis-related lncRNAs as key regulators of immune cell infiltration and patient survival, providing a novel perspective on BRCA prognosis and immunotherapy response.	The study is limited by reliance on a single public database (TCGA) and the exclusion of other potentially relevant genes and lncRNAs. Future research should incorporate additional datasets and conduct experimental validation to better understand the biological mechanisms and interactions of cuproptosis-related lncRNAs in BRCA.	Guo et al. (2022)
	YTHDF3-AS1, LINC00839, OTUD6B-AS1, NIFK-AS1, TOLLIP-AS1, TP53TG1	6	Researchers developed a comprehensive cuproptosis-related model that links lncRNAs to BRCA prognosis, enhancing predictive accuracy by integrating clinical features. This model shows promise in identifying resistance to PARP and CDK4/6 inhibitors, providing new insights into treatment strategies for BRCA.	Further validation using prospective, multicenter, real-world data and experimental studies is needed to explore the underlying molecular mechanisms of cuproptosis-related lncRNAs and their role in tumor immunity. Additional *in vitro* and *in vivo* experiments will help elucidate the interactions between these lncRNAs and immune cell infiltrations.	Wu X. et al. (2023)
	AL139241.1, MFF-DT, AL451123.1, AC009120.5, AL137847.1, HECW2-AS1, LINC01031, NIFK-AS1, AL592301.1, U73166.1	10	Novel prognostic signature (CuImP-LncRNAs) that integrates cuproptosis-related lncRNAs and immune checkpoint genes to predict breast cancer prognosis. The signature effectively stratifies patients into high-risk and low-risk groups, guiding treatment choices and offering insights into chemotherapy and immunotherapy responsiveness based on tumor immune microenvironment (TIME) and tumor mutational burden (TMB).	Further validation in diverse, multicenter cohorts and experimental studies is needed to confirm the clinical utility of the CuImP-LncRNA signature. Additionally, more in-depth exploration of its mechanisms in the context of cuproptosis and immune response is essential for optimizing personalized treatment strategies for BRCA.	Li Y. et al. (2023)

*Note. BRCA*, Breast cancer; *FI-DELs*, Ferroptosis- and Immune-related Differentially Expressed *LncRNAs*; LncRNA, Long Non-Coding RNA; *OS*, Overall survival; *TCGA*, The cancer genome atlas; *TIME*, Tumor immune microenvironment; *TMB*, Tumor mutational burden; *FRLncRNAs*, Ferroptosis-related Long Non-Coding RNAs; *RFS*, Relapse-free survival; *TNBC*, Triple-Negative Breast Cancer; *FI-DELs*, Ferroptosis and Immune-related Differentially Expressed LncRNAs; *TME*, Tumor microenvironment; *BCCuS*, Cuproptosis-related 2-lncRNA, signature; *CuImP-LncRNAs*, Cuproptosis- and Immune-related Prognostic LncRNAs; *CDK4/6*, Cyclin-dependent Kinases 4 and 6; *GEO*, Gene expression omnibus; *GSEA*, Gene set enrichment analysis.

To provide a comprehensive understanding, we briefly discuss the broader sncRNA landscape, recognizing the significant roles of miRNAs, circRNAs, and tsRNAs in cancer progression. However, the central focus remains on lncRNAs, given their pivotal role in the regulation of ferroptosis and cuproptosis in breast cancer.

### 4.1 Ferroptosis associated lncRNA in the prognosis of breast cancer

Recent research has identified eleven long non-coding RNAs (lncRNAs) associated with ferroptosis as potential prognostic indicators in breast cancer patients. Specifically, Kaplan-Meier analysis indicated that high-risk lncRNA signatures are linked to poorer outcomes. The AUC for these lncRNA signatures was 0.682, confirming their predictive accuracy for breast cancer prognosis. Among these, the lncRNAs identified as independent prognostic markers include KLHDC7B-DT, AC012213.3, LIPE-AS1, SIDT1-AS1, AC009171.2, AC137630.3, HSD11B1-AS1, LINC02446, TFAP2A-AS1, AC079298.3, YTHDF3-AS1 (Jia et al., 2021). Another study pinpointed seven ferroptosis- and immune-related differentially expressed lncRNAs (FI-DELs) namely AL035661.1, ADAMTS9-AS1, AC078883.1, FTX, AC007686.3, CBR3-AS1, TMEM105, that are significantly associated with overall survival in patients with breast infiltrating ductal and lobular carcinoma. The model had an AUC exceeding 0.6 across the training, validation, and cohorts. Furthermore, the predictive model exhibited high sensitivity (87.84%) and specificity (97.06%), underscoring its potential clinical utility (Wei et al., 2022). Moreover, a prognostic signature comprising eight ferroptosis-related lncRNAs (AL133467.1, LINC01235, AC072039.2, USP30-AS1, AC108474.1, MAPT-AS1, TDRKH-AS1, LIPE-AS1) was developed using multivariate Cox regression analysis. The predictive accuracy of this signature was validated via receiver operating characteristic (ROC) curve analysis. The area under the time-dependent ROC curve (AUC) in the training cohort was 0.853 at 1 year, 0.802 at 2 years, and 0.740 at 5 years. In the validation cohort, the AUC values were 0.791 at 1 year, 0.778 at 2 years, and 0.722 at 5 years (Zhang K. et al., 2021).

In addition to these findings, 12-FRLncRNA signature, consisting of LINC01871, LINC00393, AC121247.2, LINC02384, LIPE-AS1, HSD11B1-AS1, AC010655.2, LINC01419, PTPRD-AS1, AC099329.2, OTUD6B-AS1, and LINC02266, has been shown to predict the prognosis of breast cancer patients accurately. Research suggests this ferroptosis-related prognostic signature could be a novel biomarker for forecasting breast cancer outcomes (Xu Z. et al., 2021). Furthermore, another study identified four ferroptosis-related differentially expressed lncRNAs (FR-DELs), namely LINC01152, AC004585.1, MAPT-IT1, and AC026401.3—that are correlated with overall survival in patients with breast cancer. The AUC of the prognostic model using these four biomarkers exceeded 0.60 in all three groups. The predictive model demonstrated a sensitivity of 86.89% and a specificity of 86.73% using these biomarkers (Yao et al., 2022). Moreover, another ferroptosis-related lncRNA risk model demonstrated considerable clinical significance in predicting breast cancer prognosis and response to immunotherapy. The constructed signature could also be used to assess the immune landscape of breast cancer patients. Notably, low-risk patients exhibited enrichment of immune-related pathways and increased infiltration of various immune cell types. CYTOR, LMNTD2-AS1, LYPLAL1-AS1, USP30-AS1, RHPN1, LINC01655, AP005131.2, AC004988.1, and AC079289.3 were upregulated in a breast cancer cell line (SKBR3) compared to a normal human breast epithelial cell line (MCF10A). Conversely, HSD11B1-AS1 was downregulated in breast cancer cell lines (MCF7, SKBR3, and MDA-MB-231). Researchers have expanded upon this model to develop a hybrid nomogram capable of predicting 1-year, 3-year, and 5-year OS rates (Shen S. et al., 2022).

In addition, five lncRNAs (LINC01235, LINC02166, AL133467.1, TGFB2-AS1, and LINC02266) were found to be associated with ferroptosis, with moderate accuracy in predicting recurrence-free survival. These lncRNAs were identified as independent predictor factors, forming a nomogram for clinical RFS (Wang Y. et al., 2022). Finally, high levels of PTPRG-AS1 were detected in TNBC patients. Significantly, *POU2F2* was identified as a transcriptional activator of PTPRG-AS1, which in turn regulated ferroptosis and cell proliferation in TNBC through the miR-376c-3p/SLC7A11 signaling pathway. Consequently, the POU2F2/PTPRG-AS1/miR-376c-3p/SLC7A11 axis holds potential as both a novel biomarker and therapeutic target for ferroptosis-mediated cancer therapy in TNBC (Li J. et al., 2024).

### 4.2 Cuproptosis associated lncRNAs in the prognosis of breast cancer

Long noncoding RNAs (lncRNAs) are closely associated with the accumulation of copper ions (Scheurer et al., 2022). Notably, lncRNAs related to cuproptosis have been identified as prognostic markers for sarcoma, gastric cancer, and renal cell carcinoma (Feng A. et al., 2022; Xu S. et al., 2022; Yang M. et al., 2022). Additionally, lncRNAs are pivotal in modulating the biological processes involved in breast cancer (Jiang Z. R. et al., 2022; Pardini and Dragomir, 2021). To further illustrate, researchers have constructed prognostic biomarker/signature models that can independently predict the prognosis of breast cancer patients and estimate OS and treatment outcomes. Specifically, researchers constructed a risk model consisting of 11 cuproptosis-related lncRNAs: GORAB-AS1, AC079922.2, AL589765.4, AC005696.4, CYTOR, ZNF197-AS1, AC002398.1, AL451085.3, YTHDF3-AS1, AC008771.1, and LINC02446. The AUC values for the receiver operating characteristic (ROC) curves at 1, 3, and 5 years were 0.849, 0.779, and 0.794, respectively. Moreover, high-risk patients exhibit high sensitivity to anti-CD276 immunotherapy and conventional chemotherapeutic drugs such as imatinib, lapatinib, and pazopanib (Jiang Z. R. et al., 2022). Another recent study explored the cuproptosis-related prognostic 2-lncRNAs (USP2- AS1, NIFK-AS1) signature (BCCuS) in breast cancer and validated it as an independent prognostic factor for breast cancer (Xu Q. T. et al., 2022). Interestingly, USP2-AS1 exhibited a positive correlation with four genes *(DLAT, PDHA1, FDX1,* and *DLD)* and a negative correlation with two genes *(LIAS* and *PDHB)*. Conversely, NIFK-AS1 showed a positive correlation with three genes *(LIAS, LIPT1,* and *PDHB)* and a negative correlation with two genes *(DLD* and *DLAT)* (Xu Q. T. et al., 2022).

Ten cuproptosis-related lncRNAs have been identified as potential biomarkers for predicting the survival prognosis of breast cancer. These lncRNAs included AL118556.1, AL451123.1, MFF-DT, AL133243.2, ZKSCAN7-AS1, AC012676.3, AC009506.1, AC079766.1, MIR1915HG, and AC138028.2, which are significantly associated with OS. The study showed that the expressions of MFF-DT, AL133243.2, MIR1915HG, ZKSCAN7-AS1, and AC009506.1 were upregulated in breast cancer tissues, while AL118556.1, AL451123.1, and AC138028.2 were downregulated. Furthermore, increased expression levels of MFF-DT, AL133243.2, MIR1915HG, and ZKSCAN7-AS1, along with decreased expression levels of AL118556.1 and AC138028.2, were also observed in breast cancer cell lines. Thus, MFF-DT, AL133243.2, and MIR1915HG are anticipated to be promising prognostic markers for breast cancer (Pan et al., 2023).

In addition, nine cuproptosis-associated lncRNAs were identified, and a lncRNA–mRNA co-expression network was established. Among these, ARHGAP28-AS1, LINC01711, LRRC8C-DT, PCAT18, and SIAH2-AS1 were found to be protective lncRNAs for patients with breast cancer. In contrast, TDRKH-AS1, SAMMSON, WDFY3-AS2, and LINC00393 were identified as risk factors. This study provides a foundation for exploring predictive biomarkers in breast cancer patients and contributes to a better understanding of the biological mechanisms involving cuproptosis-related lncRNAs (Guo et al., 2022). Furthermore, researchers identified six cuproptosis-related lncRNAs that could regulate breast cancer cell proliferation and metastasis. High-risk patients have poorer survival rates and lower sensitivity to chemotherapy, endocrine therapy, and radiation therapy. Low-risk patients exhibited reduced expression of biomarkers associated with resistance to CDK4/6 inhibitors *(CCNE1, E2F1, E2F2)* and PARP inhibitors *(BRCA1/BRCA2)*, suggesting an enhanced potential for response to PARP and CDK4/6 inhibitor therapies. The plate colony formation assay showed decreased colony formation in MCF-7 cells after silencing YTHDF3-AS1, LINC00839, and OTUD6B-AS1 and increased colony formation after silencing NIFK-AS1 and TOLLIP-AS1. Similar results were observed in the CCK-8 kit assay, indicating the importance of YTHDF3-AS1, LINC00839, and OTUD6B-AS1 in promoting breast cancer cell proliferation. Knocking down YTHDF3-AS1, LINC00839, and OTUD6B-AS1 decreased MCF-7 cell invasiveness while silencing NIFK-AS1, TP53TG1, and TOLLIP-AS1 increased invasiveness. Wound healing assays yielded consistent results, suggesting that carcinogenesis-related lncRNAs play a significant role in breast cancer metastasis (Wu X. et al., 2023).

Ten long noncoding RNAs (lncRNAs), termed CuImP-LncRNAs, were identified as being associated with both cuproptosis and immune responses. These included AL139241.1, MFF-DT, AL451123.1, AC009120.5, AL137847.1, HECW2-AS1, LINC01031, NIFK-AS1, AL592301.1, and U73166.1 (Li Y. et al., 2023). Furthermore, the findings provide a novel predictive model for breast cancer prognosis, aiding in the optimization of individualized therapy for patients. This model not only offers accuracy but also opens avenues for alternative treatment approaches. In addition, this study lays the groundwork for further research into cuproptosis-related ncRNAs in breast cancer, facilitating the development of new biomarkers and therapeutic targets for this disease. Moreover, a recent study investigated nine lncRNAs related to cuproptosis (Li F. et al., 2024). Specifically, the study analyzed immune function, tumor mutation burden, and tumor immune dysfunction and exclusion differences among patients with varying risk scores, resulting in the construction of a prognostic model for breast cancer prediction. The AUC values of this model at 1 year, 3 years, and 10 years were 0.783, 0.728, and 0.795, respectively. Notably, these values surpassed those of other models, indicating its superior predictive performance (Wang F. et al., 2020; Zhang D. et al., 2020).

## 5 Potential interplay and clinical association between F/CRGs

A recent study identified potential interconnections between cuproptosis and ferroptosis regulators (Shen Y. et al., 2022). To validate the regulation between cuproptosis and ferroptosis regulators, the expression of cuproptosis regulators was analyzed by knocking down several ferroptosis regulators. In the GSE120472 cohort, knockout of *PTEN* in primary mouse embryonic fibroblasts (MEFs) resulted in the upregulation of 3 cuproptosis regulators, including *DBT, SLC31A1,* and *ATP7A*. In the GSE184356 cohort, the downregulation of *TFAM* led to a significant change in *PDHA1, PDHB, ATP7A,* and *ATP7B* in human dermal fibroblasts. In the GSE145548 cohort, the downregulation of *ATF2* in MCF7 breast cancer cells resulted in substantial changes in the expression of cuproptosis regulators *(DLST, GCSH, PDHA1, LIPT1,* and *DLD)* (Shen Y. et al., 2022). This study validated the correlation between cuproptosis and ferroptosis regulators by transfecting siRNAs and shRNA into A549 cells. The findings further revealed that *SLC31A1* expression was upregulated following *PTEN* downregulation, *ATP7A* expression increased after *TFAM* downregulation, and *LIPT1* expression was inhibited following *ATF2* downregulation. Thus, the results indicated the cross-talk and biological regulation between cuproptosis and ferroptosis regulators in cancers, including breast cancer. Moreover, a study devised a novel approach to construct a signature model comprising eleven cuproptosis-related ferroptosis genes (Zhang and Zhang, 2023). This signature included *G6PD, GPX4, PANX1, PIK3CA, CHAC1, SOCS1, CHMP6, ANO6, CS, SLC7A5,* and *EMC2,* encompassing both ferroptosis driver genes *(ANO6, CHAC1, CS, EMC2, G6PD, PANX1,* and *PIK3CA)* and ferroptosis suppressor genes *(CHMP6, GPX4,* and *SOCS1)*. The identified cuproptosis-related FRG signature shows potential as a novel prognostic biomarker for predicting overall survival in breast cancer patients (Zhang and Zhang, 2023).

**FIGURE 6:**
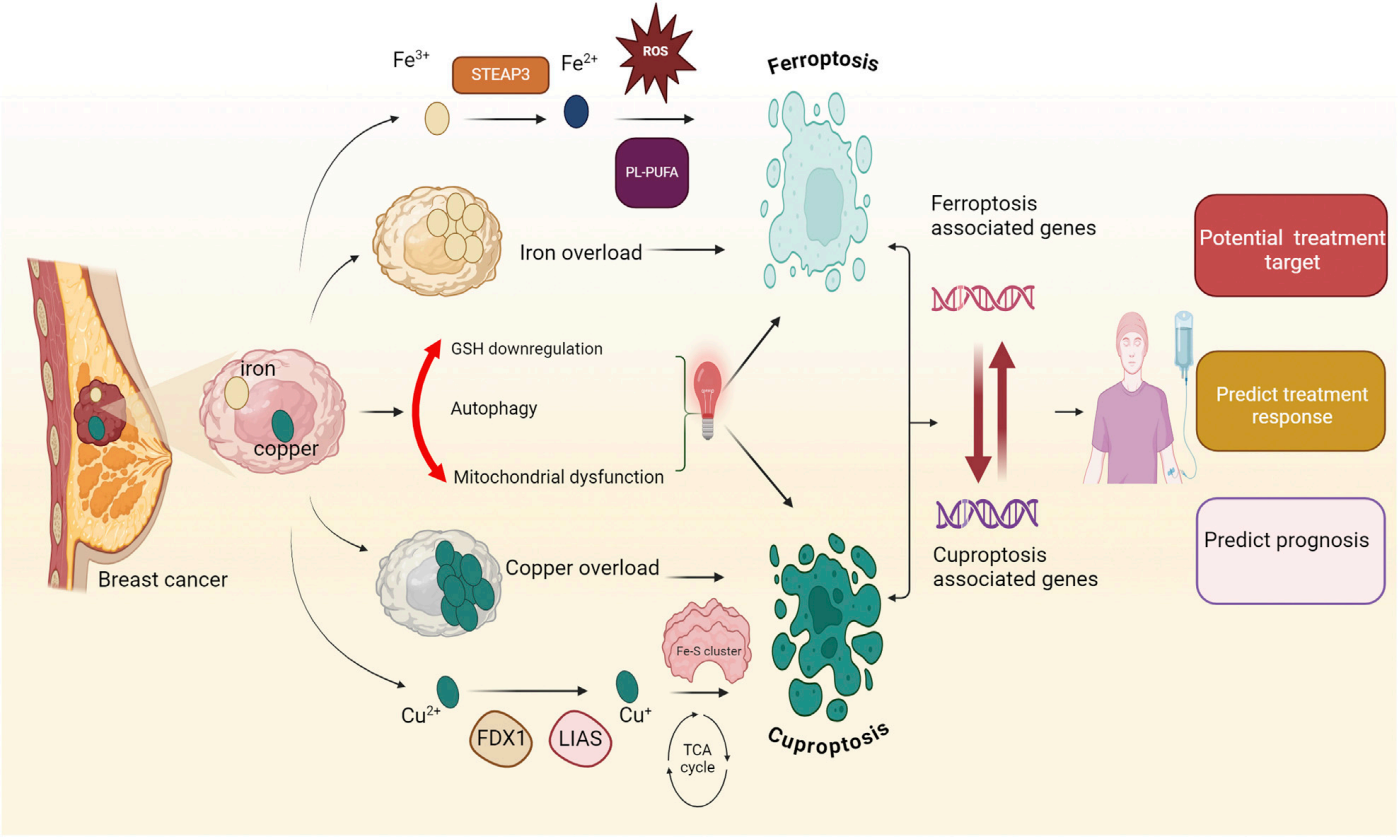
Association between ferroptosis and cuproptosis. Ferroptosis and cuproptosis are distinct forms of regulated cell death induced by iron and copper overload. Ferroptosis is characterized by the accumulation of iron-dependent lipid peroxides, leading to oxidative damage and cell death. In contrast, cuproptosis is marked by mitochondrial dysfunction and proteotoxic stress from excessive intracellular copper. The intersecting pathways of ferroptosis and cuproptosis in breast cancer highlight a complex relationship involving metal homeostasis, mitochondrial dysfunction, glutathione metabolism, and autophagy. The prognostic significance of ferroptosis and cuproptosis gene signatures in breast cancer is substantial, suggesting their potential utility in predicting survival rates, modulating treatment strategies, and addressing drug resistance challenges. The intricate interplay between ferroptosis and cuproptosis gene signatures has profound implications for both the prognosis and treatment of breast cancer. The red bidirectional arrow demonstrates an innovative linkage between ferroptosis and cuproptosis genes. The identification of this linkage suggests avenues for further investigation into the synergistic effects and potential therapeutic implications of targeting both ferroptosis and the cuproptosis pathway in breast cancer. Fe-S cluster, iron-Sulfur cluster; Fe^3+^, ferric ion; Fe^2+^, ferrous ion; Cu^2+,^ cupric ion; Cu^+^, cuprous ion; STEAP3, six-Transmembrane Epithelial Antigen of the Prostate 3; ROS, reactive Oxygen Species; PL-PUFA, phospholipid Polyunsaturated Fatty Acid; TCA cycle, tricarboxylic Acid cycle.

Moreover, another recent study revealed that the correlation between immune cell infiltration and patient outcomes in breast cancer is associated with genes linked to cuproptosis and ferroptosis (Li Y. et al., 2023). An innovative approach was employed to establish a predictive model for breast cancer patients by integrating ferroptosis and cuproptosis genes. The selected genes included *TRIM45, KNOP1, HOXC10, SGPP1,* and *ANKRD52.* The study confirmed their varied expression patterns in tumor and non-tumor biological tissue samples, both at the cellular and tissue levels. Subsequently, the relationship between these genes and tumor staging, cellular infiltration, and clinical indicators was analyzed (Li et al., 2023a). The proposed scoring model demonstrates the potential for guiding clinical decisions and tailoring treatment approaches for breast cancer patients, offering insights into selecting antitumor drugs for breast cancer treatment. As reported, a high ACSL4/low GPX4 profile holds significant practical value in predicting pathological complete response to neoadjuvant chemotherapy in patients with breast cancer (Sha et al., 2021). Future research should focus on unraveling the complex molecular mechanisms underlying the interplay between cuproptosis and ferroptosis in breast cancer. Efforts should also prioritize exploring and refining the identified signature models by incorporating additional genetic and epigenetic factors. Such advancements aim to enhance the predictive accuracy of these models, particularly in forecasting treatment responses and facilitating the personalization of therapeutic strategies (Figure 6).

### 5.1 Ferroptosis in the clinical settings of breast cancer

Ferroptosis has become a pivotal focus in cancer research due to its association with intracellular iron accumulation and disruptions in iron, lipid, and amino acid metabolism. Compounds such as erastin, RSL3, and ferrostatin-1 have been identified as key regulators of ferroptosis; however, the detailed mechanisms governing its role in cancer progression remain elusive, necessitating further investigation to unlock its therapeutic potential (Huang et al., 2024). Emerging evidence suggests that ferroptosis significantly influences breast cancer tumorigenesis, progression, invasion, and drug resistance (Ge et al., 2024). Inducing ferroptosis in these cells inhibits tumor growth, offering a promising strategy for developing new treatments and overcoming drug resistance.

#### 5.1.1 Conventional therapies and ferroptosis in breast cancer

Over the past decade, research has linked ferroptosis to chemotherapy resistance in cancer, highlighting its potential as a therapeutic strategy. Key regulators of ferroptosis, including proteins and enzymes involved in iron metabolism, lipid peroxidation, and the system Xc–pathway, play critical roles in modulating chemotherapy resistance. In TNBC, anthracyclines often demonstrate limited efficacy due to chemotherapy resistance and notable side effects. Studies suggest that inducing ferroptosis may enhance the sensitivity of TNBC cells to these agents, providing a promising approach to improve therapeutic outcomes (Luo et al., 2022; Zhao J. et al., 2023). Recent advancements in research have identified several chemotherapeutic agents that can induce ferroptosis as a mechanism for their anti-cancer effects. Notable examples include cisplatin, temozolomide, orlistat, and sorafenib. These drugs exploit the ferroptotic pathway to enhance their efficacy against various cancer types, presenting a novel strategy for improving treatment outcomes (Mou et al., 2019; Yin et al., 2022). Anomanolide C has been shown to inhibit the progression and metastasis of TNBC by enhancing the ubiquitination of the GPX4 protein, leading to autophagy-dependent ferroptosis (Chen Y-M. et al., 2023). This research highlights the potential of targeting ferroptosis as a strategy to sensitize tumors to chemotherapy in future cancer therapies. Saponin formosanin C (FC), a potent ferroptosis inducer from *Paris formosana*, enhances ferritinophagy and chemosensitivity to cisplatin in TNBC cells (Chen H-C. et al., 2022), while gallium maltolate (GaM) demonstrates synergistic anti-tumor effects with cisplatin, leading to TNBC cell death by impeding cell cycle progression and proliferation (Chen H-C. et al., 2023). Propofol, traditionally an anesthetic, has been shown to inhibit TNBC cell proliferation and potentiate doxorubicin and paclitaxel’s effects through the p53-SLC7A11-GPX4 pathway (Sun C. et al., 2022; Xu et al., 2020). Additionally, SOCS1, a new target for TNBC treatment, regulates cisplatin resistance and tumor progression (Wang et al., 2023d). A recent study provides new evidence that doxorubicin (DOX) exerts its anti-cancer effects, in part, by inducing ferroptosis. A critical role for DnaJ heat shock protein family (Hsp40) member C12 (DNAJC12) in breast cancer chemotherapy resistance was identified, as DNAJC12 was shown to suppress both DOX-induced ferroptosis and apoptosis, contributing to treatment resistance. Moreover, the DNAJC12/HSP70/AKT signaling axis appears to mediate this resistance by inhibiting these cell death pathways. Additionally, the findings further support the potential of AKT inhibitors or HSP70 inhibitors in reversing this resistance mechanism. This may offer a new perspective on overcoming DOX resistance in estrogen receptor-positive (ER+) breast cancer or other molecular subtypes with high DNAJC12 expression. These results suggest that targeting this axis may enhance the efficacy of DOX by restoring sensitivity to ferroptosis and apoptosis (Hu et al., 2024). Leveraging ferroptosis to enhance the efficacy of adjuvant chemotherapies and counteract chemotherapy resistance offers a promising therapeutic approach for treating breast cancer.

Numerous studies indicate that radiotherapy can exert anticancer effects by inducing ferroptosis. Ionizing radiation (IR) initiates cell death through several mechanisms, primarily by generating ROS and upregulating *ACSL4*, which leads to lipid peroxidation and triggers ferroptosis (Lei G. et al., 2020). A nanomedicine, BZAMH (Zeng et al., 2023), based on a metal-organic framework and combining high-Z element radio-sensitization, induces both ferroptosis and apoptosis in TNBC by disrupting GSH production with L-buthionine-sulfoximine (BSO) and promoting the Fenton reaction with supplied ferrous ions (Zeng et al., 2023). Additionally, iron-saturated lactoferrin (Holo-Lf) enhances ROS generation, damages DNA, and alleviates hypoxia in TNBC cells, improving radiosensitivity through disrupted iron metabolism and promoting ferroptosis (Zhang Z et al., 2021).

The TME has an immunosuppressive effect and lipid metabolism, a key feature of the TME, is closely linked to ferroptosis through lipid peroxidation (Mao et al., 2021; Xia et al., 2021). This intricate crosstalk between ferroptosis and the TME plays a crucial role in determining immunotherapy efficacy or resistance (Tang D. et al., 2021; Wang et al., 2021). Numerous studies demonstrate that ferroptosis significantly influences the immune response by affecting the activity of immune cells (Kim et al., 2022; Liao et al., 2022). In the TME, different immune cell subtypes, such as T cells, B cells, granulocytes, and monocytes, may experience spontaneous ferroptosis, which in turn impacts the overall immune response (Xu S. et al., 2021). In recent years, immunotherapy has emerged as a promising approach for TNBC treatment, with studies indicating its potential to induce ferroptosis. In TNBC, the LAR subtype, which expresses the androgen receptor, demonstrates increased levels of oxidized phosphatidylethanolamine and alterations in glutathione metabolism, especially involving GPX4. Notably, the inhibition of GPX4 can trigger ferroptosis in this subtype (Yang F. et al., 2023). Research has shown that inhibiting GPX4 not only triggers ferroptosis in tumors but also boosts anti-tumor immunity. The combination of a GPX4 inhibitor with anti-PD1 therapy is more effective than monotherapy (Fan Y et al., 2023). Furthermore, protein arginine methyltransferase 5 (PRMT5) plays a crucial role in regulating ferroptosis, as it methylates and stabilizes Kelch-like ECH-associated protein 1 (KEAP1), a ubiquitinating enzyme that blocks the NRF2/HO-1 pathway, increasing TNBC resistance to both ferroptosis and immunotherapy (Wang et al., 2023f). This highlights PRMT5 as a potential therapeutic target, suggesting that combining immunotherapy with PRMT5 inhibitors could enhance treatment effectiveness. Additionally, a newly designed small-molecule photosensitizer, Ir(III) photosensitizer (IrFc1) (Ling et al., 2022), coupled with transferrin and ferrocene, can promote ferroptosis in TNBC cells through a self-amplifying mechanism, leading to lipid oxidation and immunogenic cell death upon irradiation. TNBC cells exhibit higher tumor-infiltrating lymphocytes (TILs) and PD-L1 levels compared to other breast cancer subtypes, and PD-L1 inhibitors like atezolizumab and pembrolizumab have demonstrated efficacy in slowing disease progression in advanced metastatic TNBC (Adams et al., 2020; Jiang et al., 2021; Zhang X. et al., 2022). However, a limited number of patients respond effectively, primarily due to drug resistance and the absence of reliable biomarkers for patient stratification. Moreover, research by Zhou et al. has identified TYRO3 as a contributor to anti-PD-L1 resistance through the inhibition of ferroptosis via the AKT/NRF2 axis, suggesting that targeting TYRO3 with receptor tyrosine kinase inhibitors to restore ferroptosis may help overcome resistance and improve survival in aggressive TNBC cases (Jiang et al., 2021). Shao and colleagues explored the heterogeneity of ferroptosis phenotypes in different TNBC subtypes. They found increased iron metabolism in the MES subtype and highlighted ferroptosis-related pathways in the LAR subtype, while the BLIS and IM subtypes showed no distinct ferroptosis characteristics. GPX4 production and ferroptosis inhibition primarily occur through androgen receptor (AR) signaling. Thus, combining GPX4 inhibitors with immune checkpoint inhibitors may offer a novel treatment strategy for tumors resembling the LAR subtype (Jiang L. et al., 2023).

#### 5.1.2 Nanotherapeutics and ferroptosis in breast cancer

Utilizing nanoparticles characterized by their small size and low toxicity provides a solution, as these particles can effectively carry ferroptosis inducers (FINs). More importantly, nanoparticles can be engineered for targeted drug delivery to tumor cells, thereby minimizing the detrimental effects on healthy cells. Employing nanoparticle-loaded FINs for targeted transport could significantly enhance the efficacy of cancer treatments (Qi X. et al., 2022). Recent research has focused on inducing ferroptosis in breast cancer through various innovative drug delivery systems. Notable advancements include folate-labeled exosomes that enhance ferroptosis by depleting glutathione and increasing ROS in MDA-MB-231 cells. Other studies have developed nanocomposites and carrier-free nano drugs that combine ferroptosis inducers with autophagy promoters, demonstrating potent anticancer effects. These approaches highlight the potential of targeted therapies to improve breast cancer treatment outcomes (Li Z. et al., 2020).

A self-assembled nanosystem comprising drug-organic-inorganic components (DFTA) demonstrates effective inhibition of ER+ breast cancer progression. This system utilizes DOX as a chemotherapeutic agent, ferric chloride (FeCl₃) as a ferroptosis inducer, and tannic acid (TA) to activate superoxide dismutase (SOD). This combination triggers a cascade reaction that generates ROS and significantly depletes GSH levels. Furthermore, integrating photothermal therapy (PT) enhances ROS production efficiency, paving the way for a synergistic approach combining chemotherapy, PT, and ferroptosis in the treatment of ER+ breast cancer (Xiong et al., 2019). Yttrium oxide nanoparticles (Y2O3-NPs) selectively induce cytotoxicity in MDA-MB-231 cells by generating oxidative stress, elevating ROS levels, causing DNA damage, and triggering apoptosis and ferroptosis through upregulation of CASP3, CASP8, and HO-1, and downregulation of BCL2 (Emad et al., 2023). The BSOandOXA@MOF-LR formulation exhibits strong tumor-suppressive properties and significantly enhances survival rates in 4T1 tumor xenograft mice. This effect is attributed to the combined action of amplified ferroptosis and the elimination of GSH, which sensitizes cells to apoptosis (Rao et al., 2023). A novel nanoprodrug (DOX@Fc-SS-ATRA NP) combining ferrocene, a differentiation inducer (ATRA), and doxorubicin, enhances TNBC therapy by promoting ferroptosis via the Fenton reaction (Wu C et al., 2023). Furthermore, a small molecule nanoprodrug delivering chemotherapeutics (CPT), Fc, and the GPX4 inhibitor RSL3 inhibits TNBC growth and metastasis through inducing apoptosis and ferroptosis, with Fc and RSL3 amplifying chemotherapy efficacy (Chen et al., 2023).

Advances in pharmacological technologies have led to the development of innovative nanomaterials for breast cancer treatment, valued for their high efficiency and safety. This also includes mitochondria-targeted nanomaterials that deliver ferroptosis-inducing drugs, offering a promising approach to improve therapeutic outcomes. A magnetic nano-photosensitizer complex CSO-SS-Cy7-Hex/SPION/Srfn, capable of accelerating redox reactions and the Fenton reaction, enhances concentration of iron and LPO with reducing GSH levels simultaneously. Through analysis of EMT-related experimental findings, it was observed that the CSO-SS-Cy7-Hex/SPION/Srfn self-assembled complex induced ferroptosis, effectively countering multidrug resistance, invasion, and metastasis in breast cancer (Sang M. et al., 2019). Simvastatin, another ferroptosis-inducing drug, also demonstrated high efficacy with the use of novel nanomaterials. By inhibiting 3-hydroxy-3-methyl-glutaryl-coenzyme A reductase, it regulates cholesterol metabolism via Fe3O4@PCBMA magnetic nanoparticles, downregulating both the mevalonate and GPX4 pathways. This induces ferroptosis in TNBC cells without causing liver or kidney toxicity. These pathways are further linked to mitochondrial glycometabolism and lipid metabolism (Yao X. et al., 2021). A Cu-tetra (4-carboxyphenyl) porphyrin chloride (Fe(III)) Cu-TCPP(Fe)-based MOF nano-system, integrated with Au nanoparticles and loaded with RSL3, blocks GSH biosynthesis by disrupting the pentose phosphate pathway, boosting RSL3-induced ferroptosis (Li K. et al., 2022). Though still in research, these nanomaterials offer promising potential for future BRCA therapies. The incorporation of various ferroptosis-inducing agents into nanoplatforms enhances anticancer efficacy through improved targeted delivery and greater tissue permeability of nanocarriers. Recent reviews highlight these advancements in nanotechnology as promising strategies for breast cancer treatment. Moreover, a recent study demonstrated that Boswellia carterii n-hexane extract (BCHE) significantly inhibited the viability of human breast cancer cells and displayed potent *in vivo* anti-breast cancer activity without notable toxicity, inducing ferroptosis by upregulating transferrin expression and intracellular Fe^2+^ levels, downregulating glutathione peroxidase 4 (GPX4), and promoting ROS mediated lipid peroxidation in both breast cancer cells and tumor-bearing mice, suggesting BCHE as a potential therapeutic candidate for ferroptosis-targeted breast cancer treatment (Xie et al., 2024). Furthermore, a study shows that oridonin (ORI), the main active component derived from the Chinese herbal plant *Rabdosia rubescens,* boosts the anti-proliferative effect of RSL3 on breast cancer cells by facilitating ferroptosis. Mechanistically, it enhanced RSL3-induced ferroptosis in breast cancer cells through activation of the JNK/Nrf2/HO-1 signaling pathway. The combination therapy of RSL3 and ORI suppressed breast cancer cell proliferation, resulting in the accumulation of lipid peroxidation products and iron ions (Ye et al., 2024). In recent years, GSK-3β has been identified as a key tumor suppressor involved in maintaining redox balance. Research by Wang et al. demonstrated that silencing GSK-3β in MDA-MB-231 cells leads to a decrease in DMT1 levels, resulting in lower intracellular ferrous iron accumulation. This reduction subsequently diminishes the sensitivity of these cells to erastin-induced ferroptosis, suggesting that GSK-3β plays a vital role in promoting ferroptosis sensitivity in breast cancer cells (Wang L. et al., 2021). Furthermore, high expression of FADS1/2, enzymes involved in PUFA biosynthesis, was associated with poor prognosis in TNBC and sensitivity to ferroptosis inducers; however, targeting FADS1/2 conferred resistance, which could be reversed by restoring PUFA levels, and inhibiting lipid droplet formation further enhanced ferroptosis, as demonstrated in preclinical models and a patient cohort (Lorito et al., 2024). Moreover, research has shown that BET inhibitors (JQ1 and I-BET151) exert anti-cancer effects in breast cancer by inducing ferroptosis, with NCOA3, as a coactivator, interacting with NR5A2 to counteract BETi-induced ferroptosis. Mechanistically, NR5A2 and NCOA3 cooperate to upregulate NRF2, a transcription factor regulating antioxidant gene expression, and inhibiting NR5A2 or NCOA3 enhances BETi’s anti-cancer effects both *in vitro* and *in vivo*. These findings suggest that targeting NR5A2/NCOA3 in combination with BET inhibitors could provide a novel therapeutic strategy for breast cancer treatment (Qiao et al., 2020).

### 5.2 Cuproptosis in the clinical settings of breast cancer

The discovery of copper-induced cellular death has revealed a new strategy for breast cancer treatment. Promising therapeutics, such as copper ionophores and copper complexes, together with advancements in nanotechnology, have demonstrated significant efficacy in breast cancer treatment (Shanbhag et al., 2021). Copper is a critical factor in the initiation and progression of malignancies, making it a promising target for breast cancer therapy. Copper and its associated proteins, including ATOX1 and CCS, play notable roles in breast cancer progression, metastasis, and poor clinical outcomes (Huffman and O'Halloran, 2001). ATOX1 regulates intracellular copper transport to key proteins such as ATP7A, ATP7B, and SOD1, while also promoting inflammatory neovascularization, wound healing, and the migration of breast cancer cells (Blockhuys and Wittung-Stafshede, 2017). Additionally, CCS facilitates copper transport and inhibits the IGF-1-mediated binding of HIF-1α to hypoxia response elements (HRE), leading to reduced VEGF production and impaired tumor angiogenesis (Seebacher et al., 2016).

A novel approach to breast cancer treatment focuses on copper metabolism, which induces cell death through copper-dependent mechanisms and disrupts copper homeostasis (Tang et al., 2024; Yang Y. et al., 2023). Copper chelators reduce bioavailability by binding copper, while copper ionophores facilitate its intracellular accumulation; these represent the primary strategies in current therapeutic initiatives. Targeting copper-dependent cell death pathways, including cuproptosis, holds potential for improving outcomes in various malignancies, including breast cancer. Recent studies have extensively investigated cuproptosis-related compounds, such as copper ionophores, chelators, and nanomedicines, which exhibit notable anti-cancer effects in breast cancer, as summarized in Table 3.

**TABLE 3:** Anti-cancer effects of compounds related to cuproptosis in breast cancer.

Compound	Drugs/Components	Cell lines	Description	Reference
Disulfiram	Cisplatin	MCF-7, SKB-R3, MDA-MB-435S	Disulfiram reduces ALDH activity and decreases the stemness of ALDH-positive breast cancer stem-like cells, while simultaneously amplifying cisplatin-induced cytotoxicity in these cells.	Yang et al. (2019)
	-	MCF-7, MDA-MB-231	Disulfiram suppresses TGF-β-induced epithelial-mesenchymal transition EMT) in breast cancer cells by inhibiting the ERK/NF-κB/Snail signaling pathway.	Han et al. (2015)
	-	BT-549, MDA-MB-231	Disulfiram boosts the efficacy of anti-PD-1 therapy TNBC by influencing PD-L1 expression. This occurs via the epigenetic reactivation of IRF7, a key regulator of immune signaling, which enhances the tumor’s responsiveness to immune checkpoint blockade therapy.	Zheng et al. (2021)
	DOX, hydrazine	MCF-7	The combination therapy of doxorubicin (DOX), disulfiram, and hydrazine produces a synergistic effect by enhancing chemosensitivity and reducing the required dose of DOX to effectively eliminate both wild-type and DOX-resistant MCF-7 breast cancer cells.	Lafi et al. (2023)
Disulfiram/Cu	-	MCF-7, MDA-MB-231	CuET inhibits tumor growth by targeting and disrupting the NPL4/p97 segregase complex, a key player in protein degradation.	Skrott et al. (2017)
	Paclitaxel	MCF7, MDA-MB-231, T47D	Disulfiram-Cu inhibits the proliferation of breast cancer stem cells and potentiates the cytotoxic effects of paclitaxel, likely through a combined mechanism of ROS generation and NF-κB pathway suppression.	Yip et al. (2011)
	-	MDA-MB-231, MCF10DCIS.com	Disulfiram-Cu induces apoptotic cell death in breast cancer cells and inhibits the growth of tumor xenografts by targeting proteasomal activity.	Chen et al. (2006)
	-	MDA-MB-231, BT20, MDA-MB-231 (PIK3CA H1047R) MDA-MB-231 (PIK3CA-E545K)	Disulfiram-Cu decreases the expression of *PTEN* and the activation of AKT in breast cancer cells. When paired with the PI3K inhibitor LY294002, it significantly suppresses tumor xenograft growth in MDA-MB-231 cells harboring PIK3CA mutations (H1047R and E545K).	Zhang et al. (2010)
	-	MDA-MB-231, 4T1	Disulfiram-Cu treatment induces apoptosis in TNBC cells by activating caspase-3 and selectively targeting cancer stem cell-like populations. These effects are associated with a significant disruption of the STAT3 signaling pathway.	Kim Y. J. et al. (2017)
	-	BT474, SKBR3	Disulfiram-Cu induces apoptosis and eradicates cancer stem-like cells in HER-2-positive breast cancer by inhibiting the HER-2/Akt signaling pathway, potentially reducing tumor growth and progression.	Kim et al. (2016)
	-	MCF-7, HT-29	Acidic pH significantly potentiates the cytotoxic effects of the disulfiram–Cu complex in breast and colon cancer cells. This enhanced toxicity is linked to alterations in cell metabolism, modulation of Akt kinase and NF-κB pathways, and elevated production of ROS.	Navrátilová et al. (2013)
	-	MDA-MB-231, Hs578T	Disulfiram–Cu inhibits migration and invasion in TNBC cells by inducing loss of focal adhesions and destabilizing the cytoskeleton, consequently reducing tumor growth and lung metastasis.	Kim Y. J. et al. (2017)
Elesclomol	-	BRCA1-mutated breast cancer cells, Basal-like breast cancer cells	BRCA1-mutated and/or basal-like breast cancer cells, characterized by compromised base-excision repair mechanisms of oxidative DNA damage, exhibit heightened sensitivity to the therapeutic effects of elesclomol, which enhances oxidative stress-induced cell death.	Alli and Ford (2012)
ZnPT	-	MDA-MB-231, HCC1806	ZnPT triggers cuproptosis by perturbing copper balance within cells and promoting the oligomerization of *DLAT*, potentially enhancing the chemosensitivity of TNBC.	Yang X. et al. (2023)
DSF@PEG/Cu-HMSNs	Disulfram, PEG, Cu2+, HMSNs	4T1	DSF@PEG/Cu-HMSNs trigger mitochondrial protein aggregation, which leads to cuproptosis by disrupting mitochondrial function and promoting copper-dependent cell death.	Wu et al. (2019)
Cu-GA NPs	Cu2+, Gallic acid, polyvinylpyrrolidone	4T1	Cu-GA nanoparticles (NPs) induce cuproptosis and apoptosis by reducing intracellular GSH levels and increasing ROS production. When combined with chemodynamic therapy (CDT), this treatment effectively suppresses tumor growth.	Zhao F. et al. (2023)
Cu2O@CuBTC-DSF@HA nanocom-posites (CCDHs)	Cu2O, Trimesic acid, disulfiram, Hyaluronic acid	4T1	CCDHs promote cuproptosis synergistically rather than inducing apoptosis, offering enhanced anti-tumor efficacy with reduced toxicity profiles.	Zhong et al. (2023)
SonoCu	Cu2+, zeolitic imidazolate framework-8, perfluorocarbon, chlorine6, O2	4T1	Combining SonoCu with sonodynamic therapy (SDT) effectively induces cuproptosis in tumor cells while sparing normal cells. This approach achieves favorable anti-tumor outcomes with strong biosafety, making it a promising strategy for targeted cancer treatment.	Chen K. et al. (2023)
HNP	DTPH, Cu2+, disulfram, hyaluronan, artemisinin	4T1	HNP depletes GSH through its abundant disulfide bonds, sensitizing cancer cells to cuproptosis. By synergistically inducing cuproptosis, ferroptosis, and apoptosis, HNP effectively suppresses tumor growth.	Xu W. et al. (2023)
CS/MTO-Cu@AMI	Mitoxantrone, Cu2+, amiloride, chondroitin sulfate	4T1	CS/MTO-Cu@AMI induces cuproptosis and mitochondrial dysfunction, activating the AMPK pathway to promote PD-L1 degradation. This nanocomplex also enhances anti-tumor immunity by stimulating the cGAS-STING pathway.	Tian et al. (2023)
CuX-P	PD-1 overexpressing T cell membrane, Mxene, Cu2+, disulfiram	4T1	Binds to PD-L1 on tumor cells; internalizes and upregulates PD-L1 expression-Induces cuproptosis. Enhances anti-tumor immune response when combined with laser treatment.	Liu T. et al. (2023)
CuMoO4 Nanodots	Cu2+, MoO42−, SDS	MCF-7, 4T1	Under prolonged photothermal therapy (PTT), CuMoO4 Nanodots effectively induce both ferroptosis and cuproptosis in tumor cells, while also stimulating an immune response through immunogenic cell death (ICD).	Zhang J. et al. (2023)
PCB	Cu-doped polypyrrole nanoparticles BPTES, platelet membrane	4T1	PCB intensifies oxidative stress, triggering *DLAT* oligomerization via CuP release, thereby inducing cuproptosis, with the GLS1 inhibitor BPTES enhancing this effect. Moreover, PCB promotes ICD, encouraging immune cell infiltration into the tumor.	Zhang N. et al. (2024)
MetaCell	Fe3+; Cu2+; 2-Aminoterephthalic acid, thermosensitive liposome	4T1	MetaCell efficiently evades immune detection, facilitates tumor penetration, and remains stable under various conditions. It promotes both cuproptosis and ferroptosis, significantly enhancing its anti-tumor effectiveness in both *in vitro* and *in vivo* settings.	Chen K. et al. (2024)
ZCProP	Zeolitic imidazole framework-90, Cu2+; prodigiosin, PEG	4T1	ZCProP delivers copper ions and prodigiosin specifically to mitochondria, leading to cell death through a combination of cuproptosis, ferroptosis, and apoptosis. This synergistic effect enhances the therapeutic potential by targeting multiple cell death pathways simultaneously.	Deng et al. (2024)
LDH/HA/5-FU nanosheets	5-FU; copper–aluminum layered double hydroxide, hyaluronic acid Drugs; 5-FU	4T1	LDH/HA/5-FU nanosheets selectively target tumor cells, facilitating the rapid release of Cu^2^⁺ and 5-FU. This dual delivery system induces apoptosis and cuproptosis in cancer cells, while simultaneously enhancing immune responses. The combination of Cu-based CDT and chemotherapy demonstrates significant potential for treating solid tumors.	Xia et al. (2024)
E-C@DOX NPs	Cu^2+^; ellagic acid, DOX, chondroitin sulfate	4T1; MCF7^Adr^	E-C@DOX NPs effectively inhibit pathways related to tumor cell stemness and survival, while leveraging Cu to disrupt mitochondria and trigger cuproptosis. This combination suppresses the ATP-dependent drug efflux mechanisms, reversing DOX resistance.	Lu et al. (2024)
D-CuxOS@Fe–MOF	Cu^2+^; Fe^3+^; D-/L-penicillamine NH2-BDC	4T1	D-CuxOS@Fe–MOF enhances oxidative stress and induces robust ferroptosis, while synergistically triggering cuproptosis, leading to a more effective cancer therapeutic response.	Hao et al. (2024)
CQG NPs	Cu^2+^; Polyvinylpyrrolidone, Gallic acid; (3-aminopropyl) triethoxysilane, GOx	4T1	CQG NPs trigger cuproptosis by releasing copper and depleting natural copper chelators in tumor cells, leading to the disruption of their antioxidant defense. This process not only remodels the immunosuppressive tumor microenvironment but also boosts immune cell infiltration and stimulates systemic immunity.	Qiao et al. (2024)
PCD@CM	NIR-II ultrasmall polymer dots; Cu^2+^; DOX; 4T1 cell membrane Drugs; DOX, aPD-L1	4T1	PCD@CM promotes cuproptosis by inducing the aggregation of lipoylated mitochondrial proteins and the depletion of iron-sulfur proteins, leading to severe proteotoxic stress. This process is further potentiated by near-infrared (NIR-II) photothermal therapy (PTT) and GSH depletion, making tumor cells more susceptible to cuproptosis. The enhanced cuproptosis subsequently activates ICD, which boosts cytotoxic T lymphocyte infiltration and strengthens the effectiveness of PD-L1-mediated immune checkpoint blockade.	Dai et al. (2024)
Cu@CDCN	Cu^2+,^ carbon photocatalyst	4T1	Cu@CDCN, by combining photocatalytic hydrogen therapy with anchored Cu^2^⁺, induces cuproptosis, leading to mitochondrial dysfunction and significant tumor growth inhibition.	Ding et al. (2023)
ECPCP	Elesclomol–Cu, cinnamaldehyde, polyethylene glycol	4T1	ECPCP substantially extends the circulation time of the elesclomol–Cu complex, promotes its accumulation in tumor sites, and activates cuproptosis. Cu^2^⁺-driven Fenton-like reactions and cinnamaldehyde-induced ROS production disrupt redox balance, triggering ICD and synergizing cuproptosis with immunotherapy.	Wu et al. (2024)
Au@MSN-Cu/PEG/DSF	Au nanorods, Cu(NO3)2, PEG, disulfiram	4T1	In combination with PTT, Au@MSN-Cu/PEG/DSF effectively induces cell apoptosis and cuproptosis, leading to tumor cell death and significant inhibition of tumor growth.	Zhou J. et al. (2023)
CJS-Cu NPs	BETA, Cu+	4T1	CJS-Cu NPs specifically trigger cuproptosis and decrease the expression of proteins linked to metastasis, effectively halting the progression of lung metastasis.	Zheng et al. (2024)
PDA-DTC/Cu NPs	Diethyldithiocarbamate, Polydopamine, Cu^2+^	4T1	PDA-DTC/Cu NPs promote cuproptosis in tumor cells by increasing intracellular copper levels, impairing mitochondrial function, and limiting ATP production, while simultaneously aiding the repolarization of TAMs to improve the tumor immune microenvironment (TIME).	Chang et al. (2024)
Cu-LDH	Layered double hydroxide, Cu^2+^	4T1	Cu-LDH nanoparticles act as lysosomal disruptors, promoting copper overload-induced cuproptosis and pyroptosis, thereby enhancing the efficacy of cancer immunotherapy.	Zhu G. et al. (2024)
Cu-DBCO/CL	Cu-DBCO; CHO; LOX-IN-3; 2,2′-PSDA	4T1	Cu-DBCO/CL initiates both cuproptosis and ferroptosis in cancer cells, promoting ICD and remodeling the extracellular matrix (ECM), which together lead to a marked reduction in tumor growth and metastasis.	Liu et al. (2024)
HA-CD@MOF NPs	Cu^2+^, DOX, hyaluronate acid	4T1	Integrating chemodynamic therapy with Cu^2+^ overload heightens oxidative stress and mitochondrial dysfunction, thereby amplifying sensitivity to cuproptosis. HA-CD@MOF nanoparticles robustly initiate ICD and suppress tumor metastasis, thereby enhancing both anti-tumor response and immune activation.	Du et al. (2024)
M@HMnO2-DP	HMnO2, disulfram, Prodrugs 4 and 5, 4T1 cancer cell membrane	4T1	M@HMnO2-DP selectively targets tumor cells to deliver disulfiram, inducing cuproptosis without the need for external copper sources. The compound also disrupts glycolytic pathways and interferes with Fe–S protein synthesis, thus enhancing sensitivity to cuproptosis.	Zhu J. et al. (2024)
PCM nanoinducers	PEG-polyphenol-Ce6 polymer Cu^2+^; Mdivi-1	4T1	PCM nano-inducers intensify proteotoxic stress via cuproptosis, triggering mitochondrial DNA (mtDNA) release. This release activates the cGAS-STING pathway, prompting a robust innate and adaptive immune response that significantly inhibits tumor progression and metastasis.	Yu et al. (2024)

*Note:* GPX4, Glutathione Peroxidase 4; GSH, Glutathione; ALDH, Aldehyde dehydrogenase; EMT, Epithelial-Mesenchymal Transition; TNBC, Triple-Negative Breast Cancer; DOX, Doxorubicin; ROS, Reactive oxygen species; NPs, Nanoparticles; CDT, Chemodynamic therapy; SDT, Sonodynamic therapy; PTT, Photothermal therapy; ICD, Immunogenic cell death; ECM, Extracellular matrix; mtDNA, Mitochondrial DNA.

#### 5.2.1 Copper ionophores

Elevated intracellular copper accumulates in the mitochondria, where it interacts with lipoacylated proteins, causing the oligomerization of DLAT and destabilizing the Fe-S cluster proteins. This disruption leads to cancer cell death, termed cuproptosis (Kahlson and Dixon, 2022). This mechanism has prompted the exploration of copper ionophores, such as DSF and ES, which increase intracellular copper levels and could potentially treat breast cancer. These ionophores form lipophilic complexes with copper, enhancing its cellular accumulation, and thus showing promise for cancer therapy (Lelièvre et al., 2020).

The anti-cancer effects of disulfiram, particularly in breast cancer, have been extensively investigated. DSF, a well-known aldehyde dehydrogenase (ALDH) inhibitor, has demonstrated significant anti-cancer properties, particularly against ALDH-positive breast cancer stem cells (Ni Y. L. et al., 2022). In addition to its targeting of these stem cells, DSF enhances ROS accumulation, thereby effectively overcoming cisplatin resistance in breast cancer cell lines. Such insights may shape future chemotherapeutic strategies (Yang et al., 2019). Research conducted by Swetha et al. indicates that the combination of DSF with docetaxel-loaded nanoparticles enhances the sensitivity of drug-resistant breast cancer cells to docetaxel. This sensitization occurs through the inhibition of the drug efflux pump P-glycoprotein, as well as the targeting of cancer stem cells (CSCs) (Swetha et al., 2023). Zheng et al. demonstrated that DSF enhances PD-L1 expression in TNBC cells, significantly improving the response of mouse breast cancer models to anti-PD-1 antibody therapy. This suggests that combining DSF with anti-PD-1 therapy could be a promising strategy for enhancing TNBC treatment outcomes (Zheng et al., 2021). Furthermore, a combination therapy evident against MCF-7 breast cancer cells, has proven to have a synergistic effect of DOX with hydrazine (Hyd) and DSF to lower the dose of the chemotherapeutic drug (Lafi et al., 2023). DSF couples with copper as a copper ionophore to produce the metabolite bisdiethyldithiocarbamate-copper (CuET), facilitating the transport of copper across the cell membrane (Lu et al., 2021). In addition, cellular damage in breast cancer cell lines resulting from DSF/Cu is associated with apoptosis, ferroptosis, and cuproptosis (Table 3). DSF/Cu inhibits the proliferation of breast cancer stem cells and potentiates the cytotoxic effects of paclitaxel, likely through a combined mechanism of ROS generation and NF-κB pathway suppression (Yip et al., 2011). Acidic pH significantly enhances the cytotoxic effects of the DSF–Cu complex in breast and colon cancer cells. This enhanced toxicity is linked to alterations in cell metabolism, modulation of AKT kinase and NF-κB pathways, and elevated production of ROS (Navrátilová et al., 2013). However, the current clinical effectiveness of DSF/Cu remains ambiguous. Notably, findings from a prospective clinical trial examining DSF paired with Cu for metastatic prostate cancer revealed that DSF was rapidly converted to the inactive metabolite Me-DDC, which resulted in no observable clinical benefits (Zhang T. et al., 2022). In contrast, a Phase II trial indicated that the combination of disulfiram with cisplatin and vinorelbine led to rare long-term survival in two-stage IV lung cancer patients (Nechushtan et al., 2015). Additionally, two separate clinical trials investigating the combination of DSF and Cu for patients with liver metastases (Kelley et al., 2021) and recurrent gliomas (Werlenius et al., 2023) also failed to show significant survival advantages. These divergent results emphasize the need for further research to optimize disulfiram’s therapeutic applications and assess its efficacy across different cancer types, including breast cancer.

ES, a lipophilic copper-binding compound, is capable of chelating extracellular Cu^2^⁺ ions to form a complex that enables the efficient transport of copper into cells (Gohil, 2021; Guthrie et al., 2020). Once inside the cells, ES exerts anticancer effects primarily by targeting mitochondrial metabolism, inducing oxidative stress, and reducing the expression of CSC markers like CD133 and ALDH (Harrington et al., 2020). Additionally, recent evidence underscores its ability to induce copper-dependent cell death (Tsvetkov et al., 2019), reinforcing its role as a promising agent in breast cancer therapy (Alli and Ford, 2012). Serum lactate dehydrogenase (LDH) levels are closely linked to mitochondrial metabolism, with lower LDH levels correlating to increased sensitivity to ES (Zheng et al., 2022). This observation has significant implications for the use of LDH as a predictive biomarker in cuproptosis-targeting treatments. Notably, in a phase III clinical trial, ES combined with paclitaxel showed limited success overall but demonstrated enhanced efficacy in melanoma patients with lower LDH levels (O'Day et al., 2013), suggesting that mitochondrial metabolism plays a pivotal role in cuproptosis activation. Earlier trials, such as a phase II study, had shown that the addition of ES to paclitaxel significantly prolonged progression-free survival (PFS) in melanoma patients by 41.7%, highlighting its potential as an anti-cancer agent (O'Day et al., 2009). Furthermore, a phase I trial in patients with refractory solid tumors, including Kaposi sarcoma and ovarian cancer, provided partial responses (Berkenblit et al., 2007), further suggesting that ES copper-dependent mechanism could enhance therapeutic outcomes. However, despite promising early-phase results, ES has not consistently demonstrated robust clinical benefits. One potential reason for this is its inability to sufficiently elevate copper levels in tumor cells when used as a monotherapy. This reinforces the need for combinatory strategies and highlights the importance of biomarkers like LDH to optimize patient selection and treatment efficacy.

#### 5.2.2 Small molecular compounds and copper complex

Copper plays a complex and dual role in tumor development. Elevated Cu levels are linked to enhanced tumor cell proliferation and growth, which suggests that tumor cells may employ certain mechanisms to resist cuproptosis. Consequently, targeting Cu homeostasis with small molecular compounds offers a promising strategy to induce or heighten tumor cell sensitivity to cuproptosis. Several recent studies have identified small molecular compounds capable of inducing this copper-dependent cell death. For example, Yang et al. demonstrated that zinc pyrithione disrupts intracellular Cu balance and triggers DLAT oligomerization in TNBC cells, which may contribute to their increased chemosensitivity (Yang X. et al., 2023). Recent research highlights that ferroptosis inducers also potentiate cuproptosis in cancer cells. For example, Sorafenib, the first multi-tyrosine kinase inhibitor approved for various cancers, and erastin, a well-known ferroptosis inducer, have been shown to exacerbate cuproptosis in liver cancer cells (Wang W. et al., 2023), providing new avenues for combined therapeutic strategies. In contrast to copper ionophores, which require copper supplementation, small-molecule compounds like *ZnPT* that disrupt intracellular copper homeostasis present a more refined therapeutic approach. These compounds can induce cuproptosis in tumor cells without introducing excess copper, minimizing the risk of metal ion imbalances and reducing potential side effects associated with metal toxicity. This method highlights the potential of targeted treatments that leverage intrinsic cellular mechanisms while mitigating adverse effects during therapy.

Copper complexes exhibit diverse catalytic and electrochemical properties (Balsa et al., 2023), positioning them as promising candidates for breast cancer therapy. These complexes exert tumor-inhibitory effects by generating ROS, depleting intracellular glutathione, impairing proteasome function, and inducing DNA damage (Ji et al., 2023), all of which contribute to the selective targeting of cancer cells. Through these mechanisms, copper complexes disrupt cellular homeostasis, promoting cancer cell death and offering potential therapeutic advantages over traditional treatments. Studies have demonstrated that copper complexes like CuHL1 and Cu-PLN inhibit the growth of TNBC cells by triggering cuproptosis. CuHL1 achieves this by downregulating DLAT expression via copper-induced protein lipoylation, while Cu-PLN contributes to cancer cell death by generating ROS, causing DNA damage, and disrupting mitochondrial function, ultimately affecting cellular metabolism (Mukherjee et al., 2023). The study by Xu et al. explored a copper-based complex called HA-Cu, synthesized from disulfonamide-dimethylpyrimidine-phenanthroline-metal, which showed potent inhibitory effects on TNBC (Xu et al., 2024). This complex was effective both *in vitro* and *in vivo*, suppressing tumor growth through a synergistic mechanism. It combined antiproliferative, antiangiogenic, anti-inflammatory, and pro-apoptotic properties, inducing cuproptosis in MDA-MB-231 cells by reducing FDX1 expression and increasing HSP70 expression (Xu et al., 2024). Similarly, He et al. developed a copper-chelated cyanine dye to deliver copper ions in various oxidation states, which were tested in 4T1 cells and 4T1 tumor-bearing mice (He et al., 2024). This approach further explored the role of copper in combating TNBC, reinforcing the idea that copper, through various mechanisms like cuproptosis and regulation of key cellular pathways, can be a promising strategy in breast cancer treatment​. These mechanisms highlight the potential of copper complexes as targeted therapies against aggressive cancers like breast cancer.

#### 5.2.3 Nano-based therapeutics

Over the past 3 decades, there has been a significant expansion of research in the field of cancer nanotherapeutics (Fan D. et al., 2023). Drugs can be transformed into nanotherapeutics through processes such as dissolution, adsorption, encapsulation, or binding to nanomaterials. These nanotherapeutics leverage unique tumor characteristics such as acidic microenvironments, high levels of GSH and ROS, or tumor-specific surface markers to achieve targeted accumulation and controlled release at the tumor site. This strategic targeting enhances therapeutic precision, significantly reducing off-target effects and the toxicity associated with conventional cancer treatments. The therapeutic effectiveness of chemotherapy can be further improved by combining it with nanocarrier-based methods like photodynamic therapy (PDT), photothermal therapy (PTT), or chemodynamic therapy (CDT), enhancing the overall anticancer efficacy. CDT leverages the Fenton reaction to generate hydroxyl radicals (⋅OH) from overexpressed H₂O₂ within tumor cells, effectively inducing oxidative stress and cell death without the need for external energy sources (Zhao F. et al., 2023).

Given the relatively low selectivity of copper ionophores for tumor cells, employing nanoparticle-based delivery systems can more effectively direct copper to tumor tissues (Zhang C. et al., 2024). This approach heightens cuproptosis activity within tumors while minimizing harm to healthy tissues. Since the identification of cuproptosis, research has increasingly focused on its potential therapeutic applications, particularly in nanoparticle-facilitated copper delivery systems. Researchers have focused on precisely delivering copper, copper ionophores, and other anticancer agents like chemotherapeutic drugs and siRNA to enhance nanomedicine-induced cuproptosis. This targeted approach synergistically increases breast tumor cell damage by sensitizing them to cuproptosis while simultaneously activating additional therapeutic mechanisms (Table 3).

Cu-GA nanoparticles (NPs), which are composed of Cu^2^⁺, gallic acid, and polyvinylpyrrolidone, have been shown to induce cuproptosis and apoptosis by depleting intracellular GSH levels and increasing ROS production. When combined with CDT, this approach significantly suppresses tumor growth, demonstrating a synergistic effect that enhances therapeutic efficacy (Zhao F. et al., 2023). Similarly, Cu2O@CuBTC-DSF@HA nanocomposites (CCDHs), constructed from Cu₂O, trimesic acid, disulfiram, and hyaluronic acid, offer a unique therapeutic approach by promoting cuproptosis synergistically, without inducing apoptosis. This mode of action increases anti-tumor efficacy while minimizing toxicity, making it a promising strategy for breast cancer treatment (Zhong et al., 2023). SonoCu nanoparticles, a combination of Cu^2^⁺, a zeolitic imidazolate framework, perfluorocarbon, chlorine-6, and oxygen, have been integrated with sonodynamic therapy (SDT). This system induces cuproptosis specifically in tumor cells while sparing normal cells, leading to strong biosafety and favorable anti-tumor outcomes, which highlights its potential for targeted cancer therapies (Chen K. et al., 2023). HNPs, comprised of DTPH, Cu^2^⁺, disulfiram, hyaluronan, and artemisinin, also show promise. These particles deplete GSH through their disulfide bonds, sensitizing cancer cells to cuproptosis. By simultaneously inducing cuproptosis, ferroptosis, and apoptosis, HNPs can effectively suppress tumor growth, particularly in 4T1 breast cancer model (Xu W. et al., 2023). CS/MTO-Cu@AMI, a nanocomplex consisting of mitoxantrone, Cu^2^⁺, amiloride, and chondroitin sulfate, induces cuproptosis and disrupts mitochondrial function in 4T1 breast cancer tumors. By activating the AMPK pathway, this formulation promotes PD-L1 degradation, effectively reducing immune evasion by cancer cells. Furthermore, CS/MTO-Cu@AMI enhances anti-tumor immunity by stimulating the cGAS-STING pathway, which plays a critical role in promoting immune surveillance and response against tumors, making it a promising candidate for combination therapies targeting immune checkpoints (Tian et al., 2023). CuX-P binds to PD-L1 on tumor cells, leading to its internalization, and subsequently triggering cuproptosis. When combined with laser treatment, this process amplifies the anti-tumor immune response by enhancing immune system activation (Liu T. et al., 2023). CuMoO4 Nanodots use prolonged photothermal therapy (PTT) to simultaneously induce ferroptosis and cuproptosis in 4T1 and MCF-7 tumors. This dual-action approach stimulates an immune response through immunogenic cell death (ICD), enhancing its effectiveness as a therapeutic strategy (Zhang J. et al., 2023). ZCProP specifically targets mitochondria, delivering copper ions and prodigiosin, which synergistically induce cuproptosis, ferroptosis, and apoptosis (Deng et al., 2024). The LDH/HA/5-FU nanosheets are designed to selectively target tumor cells, facilitating the rapid release of Cu^2^⁺ and 5-FU, which together induce apoptosis and cuproptosis while enhancing immune responses. This dual delivery system represents a promising approach that combines Cu-based CDT with chemotherapy for solid tumor treatment (Xia et al., 2024). Complementing this, E-C@DOX NPs effectively inhibit tumor cell stemness and survival pathways, utilizing Cu to disrupt mitochondrial function and trigger cuproptosis, which counters ATP-dependent drug efflux mechanisms, thus reversing DOX resistance (Lu et al., 2024). Additionally, D-CuxOS@Fe–MOF enhances oxidative stress, robustly inducing both ferroptosis and cuproptosis for improved therapeutic response against cancer (Hao et al., 2024). Cu@CDCN further enhances this synergy by integrating photocatalytic hydrogen therapy with anchored Cu^2^⁺, inducing cuproptosis and mitochondrial dysfunction, ultimately inhibiting tumor cell proliferation and significantly suppressing tumor growth (Ding et al., 2023).

Moreover, ECPCP substantially extends the circulation time of the elesclomol–Cu complex, promoting its accumulation at tumor sites and activating cuproptosis. The complex’s Cu^2^⁺-driven Fenton-like reactions, alongside ROS production from cinnamaldehyde, disrupt redox balance, triggering ICD and enhancing synergy with immunotherapy (Wu et al., 2024). Furthermore, Au@MSN-Cu/PEG/DSF, CJS-Cu NPs, PDA-DTC/Cu NPs, Cu-LDH, Cu-DBCO/CL, and HA-CD@MOF NPs also represent a promising Frontier in breast cancer therapy by leveraging copper’s unique properties for targeted treatment strategies (Table 3) (Chang et al., 2024; Du et al., 2024; Liu et al., 2024; Zheng et al., 2024; Zhou J. et al., 2023; Zhu G. et al., 2024). Additional studies underscore the potential of novel cuproptosis-based nanotherapeutics in breast cancer. M@HMnO2-DP selectively targets tumor cells, delivering DSF to induce cuproptosis independently of external copper sources while disrupting glycolytic pathways and interfering with Fe–S protein synthesis (Zhu J. et al., 2024). Similarly, PCM nano-inducers enhance proteotoxic stress through cuproptosis, leading to the release of mitochondrial DNA that activates the cGAS-STING pathway, thereby initiating a robust immune response that significantly inhibits tumor progression and metastasis (Yu et al., 2024). A novel strategy involving the co-delivery of Cu and erastin in cancer cells has been developed to achieve a synergistic effect by inducing both cuproptosis and ferroptosis (Li Y. et al., 2024). Copper/erastin (CuP/Er) enhances immunogenic cell death, improves antigen presentation, and upregulates PD-L1, promoting T cell proliferation and infiltration. When combined with immune checkpoint inhibitors, it reactivates T cells, leading to effective tumor regression and preventing metastasis in colon adenocarcinoma and triple-negative breast cancer models (Li Y. et al., 2024). This approach underscores the potential of combining multiple cell death pathways to amplify the efficacy of cancer immunotherapies. Expanding on this approach, Huang et al. developed a poly (amidoamine) dendrimer modified with p-carboxybenzenesulfonamide, loaded with copper peroxide nanoparticles and combined with iron-tannic acid networks to form the nanocomposite CuO₂@G5-BS/TF (Huang et al., 2024). This nanocomposite targeted 4T1 tumor cells, facilitating MRI imaging and inducing both ferroptosis and cuproptosis by depleting glutathione and overloading copper and iron. It further neutralized the acidic tumor microenvironment, effectively inhibiting metastasis and offering a dual-functional strategy for TNBC imaging and treatment (Huang et al., 2024). Similarly, the MetaCell system demonstrated the ability to evade immune detection, penetrate breast tumor cells, and promote both cuproptosis and ferroptosis, which resulted in significant anti-tumor effects *in vitro* and *in vivo* (Chen K. et al., 2024). Utilizing these therapies, either independently or alongside conventional treatments like chemotherapy and radiotherapy, could greatly enhance breast cancer management. Continued investigation is essential to fully harness their capabilities and improve clinical applications.

### 5.3 Clinical trials targeting ferroptosis and cuproptosis in breast cancer

Recent clinical trials are investigating ferroptosis and cuproptosis modulation as promising strategies for treating breast cancer, particularly in advanced or treatment-resistant cases (ClinicalTrials.gov). In ferroptosis-targeted approaches, sorafenib, a System Xc-inhibitor, is undergoing a phase II trial in metastatic breast cancer, aiming to disrupt glutathione synthesis and thereby promote ferroptosis (NCT00101400). Similarly, artesunate, an iron-oxidizing agent, is being assessed in a phase I trial for metastatic breast cancer due to its ability to induce oxidative stress in iron-abundant tumor environments (NCT00764036). Neratinib, which activates iron-dependent pathways, is in phase II trials for both HER-2-positive (NCT00300781) and ER-positive breast cancers (NCT05933395), where it may increase susceptibility to ferroptosis in these specific subtypes. Additionally, deferoxamine (DFO), an iron chelator, is being explored in a phase II trial for metastatic triple-negative breast cancer to deplete iron stores and selectively trigger ferroptotic cell death in aggressive tumor cells (NCT05300958).

Copper modulation is also under investigation, showing promise for advanced breast cancer cases. Disulfiram combined with copper supplementation is in phase II trials for metastatic breast cancer that has progressed despite conventional systemic and/or locoregional therapies (NCT03323346), as well as for CTC_EMT-positive, refractory, metastatic hormone receptor-positive, HER-2-negative breast cancer (NCT04265274). Copper depletion strategies are being explored with ATN-224, in a phase II trial for recurrent or advanced estrogen and progesterone receptor-positive breast cancer (NCT00674557). Tetrathiomolybdate, another copper-depleting agent, is under investigation for various breast cancer risk categories: one phase II trial assesses its role in breast cancer with moderate-to-high recurrence risk (NCT00195091), while a phase I/II trial evaluates its effectiveness in reducing relapse risk in high-risk, TNBC (NCT06134375). These trials collectively seek to delineate the therapeutic potential of ferroptosis and cuproptosis modulation, aiming to improve outcomes and overcome resistance in challenging breast cancer subtypes.

## 6 Pros and cons of ferroptosis and cuproptosis in breast cancer

Breast cancer displays considerable heterogeneity, not only across different patients but also within tumors from the same individual. This diversity is largely defined by differences in hormone receptor status, HER-2 expression, genetic mutations, and the varying characteristics found within individual tumors (Liu S. Q. et al., 2022; Taylor et al., 2024). Hormone receptor-positive breast cancers (ER+ and/or PR+) and TNBC exhibit significant differences in their biology, response to treatments, and overall prognosis (Amgad et al., 2024). Tumors with HER-2 overexpression respond well to therapies specifically targeting HER-2, such as trastuzumab, but may show diminished effectiveness with non-targeted treatment options (Sirhan et al., 2022). Frequent genetic mutations in breast cancer, such as *TP53*, *PIK3CA*, and *GATA3*, play a key role in shaping tumor aggressiveness and influencing treatment outcomes (Martínez-Sáez et al., 2020; Mosele et al., 2020). Furthermore, breast tumors often exhibit intra-tumoral heterogeneity, with distinct cellular subpopulations displaying differences in morphology, growth potential, and gene expression (Martelotto et al., 2014). This molecular diversity drives the tumor’s biological characteristics and significantly affects how it responds to different treatment approaches.

### 6.1 Advantages

Inducing ferroptosis and cuproptosis represents a compelling therapeutic strategy for targeting breast cancer’s metabolic weaknesses, particularly in treatment-resistant cases. Ferroptosis capitalizes on cancer cells’ iron dependency and susceptibility to lipid peroxidation, leading to oxidative damage and cell death. On the other hand, cuproptosis leverages the toxic accumulation of copper to disrupt mitochondrial function, causing cell death by impairing energy production. These distinct pathways exploit specific vulnerabilities in aggressive breast cancer subtypes, such as TNBC, significantly enhancing the potential for cancer cell eradication. Both paths offer promising solutions for overcoming treatment resistance, a common challenge in breast cancer therapy. Many cancer cells that develop resistance to conventional treatments such as chemotherapy, radiotherapy, or hormonal therapy remain susceptible to ferroptosis and cuproptosis. This opens new avenues for therapy, potentially restoring the effectiveness of existing treatments. Additionally, the selective nature of these cell death mechanisms, which rely on the disruption of iron and copper homeostasis, makes them more likely to affect cancer cells preferentially, thereby minimizing collateral damage to healthy tissues. Furthermore, combining ferroptosis and cuproptosis with conventional therapies, such as chemotherapy and immunotherapy, may result in synergistic effects. By simultaneously targeting cancer cells through disruption of both iron and copper metabolism, along with traditional cytotoxic therapies, the overall therapeutic efficacy can be enhanced. Preclinical studies have shown that ferroptosis not only inhibits tumor growth but also suppresses metastasis in breast cancer models. Although cuproptosis is a newer concept, its disruption of mitochondrial function offers a similar potential for tumor metastasis, particularly in highly proliferative cancer cells. Beyond their cytotoxic effects, ferroptosis has been associated with immunogenic cell death, which may enhance the efficacy of immunotherapies. Similarly, there is growing interest in the potential of cuproptosis to stimulate an immune response, thereby further increasing its therapeutic value. Both pathways demonstrate versatility across breast cancer subtypes, including TNBC and HER-2-positive cancers, by addressing distinct metabolic vulnerabilities—ferroptosis targeting iron metabolism and cuproptosis focusing on mitochondrial activity and copper homeostasis. This versatility highlights their potential as powerful tools in the fight against breast cancer cases (Ai L. et al., 2024; Li Z. et al., 2020; Qi X. et al., 2022; Zhang C. et al., 2024; Zhou et al., 2022; Zhu Z. et al., 2024).

By targeting these regulated cell death pathways, researchers and clinicians can address the heterogeneity of breast cancer, offering new treatment options for resistant or aggressive forms of the disease.

### 6.2 Disadvantages

Inducing ferroptosis and cuproptosis in breast cancer represents several challenges. Off-target toxicity is a primary concern, as disrupting iron or copper homeostasis could harm healthy tissues, particularly vital organs like the liver and brain, especially with GPX4 inhibitors affecting the development and function of the nervous system and kidneys (Koppula et al., 2021; Liang et al., 2019). This could lead to organ dysfunction and systemic toxicity. Additionally, inflammatory responses triggered by ferroptosis-related lipid peroxidation or cuproptosis-related mitochondrial dysfunction may exacerbate inflammation, potentially promoting tumor progression or treatment resistance. Tumor heterogeneity adds complexity, as cancer cells vary in their susceptibility to ferroptosis (Liang et al., 2019) and cuproptosis, allowing some cells to evade death or develop resistance by enhancing antioxidant defenses or modulating metal metabolism. Moreover, the mechanistic understanding of these pathways *in vivo* remains incomplete, which could result in unexpected toxicities or interactions with other cell death mechanisms like apoptosis or autophagy. Targeting specificity is another challenge, as developing therapies that induce ferroptosis or cuproptosis selectively in cancer cells without affecting healthy tissues is difficult, increasing the risk of broad cytotoxic effects. Unpredictable interactions with existing treatments, such as chemotherapy, radiotherapy, or immunotherapy, may complicate treatment outcomes and limit their effectiveness. Lastly, prolonged targeting of metal metabolism risks systemic dysregulation, potentially leading to iron overload or copper toxicity, which could cause long-term tissue damage (Chen Z. et al., 2023; Kciuk et al., 2024; Zhou Q. et al., 2024). Addressing these challenges is critical to safely harnessing ferroptosis and cuproptosis for breast cancer treatment.

Ferroptosis and cuproptosis inducers show potential in enhancing chemotherapy effects and overcoming drug resistance in cancer treatment. However, combining these therapies can cause side effects on normal tissues. To address this, there is a need for a drug that co-targets both pathways, responding selectively to conditions in the tumor microenvironment, while minimizing harm to healthy cells. Developing such a compound is challenging and requires careful consideration of various factors, including tumor heterogeneity and potential toxicity.

## 7 Overview and perspectives

Breast cancer treatment encompasses multiple modalities, including surgery, chemotherapy, radiotherapy, hormone therapy, targeted therapy, and immunotherapy. Recent advances underscore the potential of ferroptosis and cuproptosis to enhance the efficacy of these treatments, particularly in overcoming therapeutic resistance. Chemotherapy remains a cornerstone of treatment for metastatic breast cancer; however, it frequently encounters resistance. The introduction of ferroptosis inducers has shown promise in sensitizing breast cancer cells, especially in TNBC. For instance, combining ferroptosis inducers with anthracyclines (Zhao J. et al., 2023) may enhance therapeutic responses by reversing drug resistance (Zhao J. et al., 2023). In the context of multiple myeloma, the activation of ferroptosis through erastin has been demonstrated to synergize with DOX, thereby enhancing its anti-cancer efficacy (Fu et al., 2022). Nevertheless, a major obstacle remains in inducing ferroptosis to combat anthracycline resistance while minimizing the risk of cardiac toxicity, which continues to be a critical area of research focus. In a similar vein, cuproptosis also contributes to tumor suppression in cancer (Tong et al., 2022). In colorectal cancer, curcumin functions as a copper ionophore, facilitating cuproptosis to exert anti-cancer effects (Yang Y. et al., 2022). Moreover, the combination of ES and 4-octyl itaconate (4-OI) has demonstrated a significant reduction in cell proliferation in oxaliplatin-resistant colorectal cells, primarily through the promotion of cuproptosis via GAPDH inhibition and suppression of aerobic glycolysis (Yang et al., 2023). Additionally, ES has been shown to elevate ROS levels in cisplatin-resistant lung cancer cells, aiding in the eradication of drug-resistant tumor cells (Tong et al., 2022). Lun et al. found that combining low doses of DSF with copper supplementation significantly enhances the effectiveness of temozolomide (TMZ) *in vitro*. This combination therapy also resulted in improved survival rates *in vivo* within brain tumor-initiating cell (BTIC) models derived from patients, which are typically resistant to standard treatments and come from both newly diagnosed and recurrent tumors. Thus, the DSF-Cu combination holds potential as an adjuvant therapy for glioblastoma management at initial diagnosis and during recurrence (Lun et al., 2016). In addition, cuproptosis can also be targeted in breast cancer; TNBC’s heightened copper demand allows for the use of copper ionophores, which induce cuproptosis and promote mitochondrial stress, offering a complementary strategy to chemotherapeutics. We have compiled the findings on cuproptosis and its relevance to breast cancer in the previous sections and Table 3.

Radiotherapy, typically utilized postoperatively to eliminate residual cancer cells and reduce recurrence risk, may benefit from the induction of ferroptosis (Lang et al., 2019; Lei G. et al., 2020). Resistance to radiotherapy is a significant factor contributing to its limited effectiveness. The sensitivity of tumors to radiotherapy can differ not only between various tumor types but also among individuals with the same diagnosis, likely due to tumor heterogeneity (Gong et al., 2021; Schaue and McBride, 2015). Recent research into radiotherapy resistance has indicated that ferroptosis inducers (FINs) can enhance the sensitivity of ionizing radiation-resistant cells and xenograft tumors to radiation therapy. Compounds such as erastin, sulfasalazine, FIN56, and RSL3 have demonstrated potential in this regard (Lei G. et al., 2020; Ye et al., 2020; Zhang W. et al., 2021). There is optimism for the development of effective combined therapies that incorporate these drugs with radiotherapy for improved cancer treatment in clinical settings. Moreover, integrating copper-mediated cell death with radiotherapy and chemotherapy has the potential to enhance their anti-cancer effects. Cancer stem cells (CSCs) are involved in treatment resistance (Zhang L. et al., 2023), and their high mitochondrial metabolism makes cuproptosis an appealing strategy to overcome this challenge (Ni et al., 2022). A recent study by Wang et al. demonstrated that combining ionizing radiation with DSF and Cu induced a robust cell stress response in breast cancer. This approach significantly increased immunogenic cell death in both differentiated cancer cells and CSCs, while also promoting the expansion of chimeric antigen receptor T cells (CAR-T) (Wang Y. et al., 2023c), a cutting-edge immunotherapy. Consequently, DSF/Cu may enhance the effectiveness of CAR-T therapy, offering promising new treatment options for breast cancer. It is well-established that immunotherapies have shown great efficacy against numerous tumors. However, resistance to immunotherapies remains a challenge due to low immune cell infiltration or suppression of anti-tumor responses within the TME. The TME plays an immunosuppressive role, with lipid metabolism being a defining aspect closely tied to ferroptosis through lipid peroxidation pathways (Mao et al., 2021; Xia et al., 2021). This intricate interaction between ferroptosis and the TME significantly affects the response to immunotherapy, either enhancing effectiveness or contributing to resistance (Tang D. et al., 2020; Wang et al., 2021). Ferroptosis-targeting therapies can enhance immunotherapy efficacy by overcoming resistance, alleviating suppression of anti-tumor immune cells, and selectively inducing ferroptosis in immunosuppressive cells (Zhai et al., 2024). Cystinase, a ferroptosis inducer (FIN), significantly raises oxidative stress, promoting cancer cell death (Cramer et al., 2017). In ovarian cancer models, combining cystinase with immune checkpoint inhibitors, specifically PD-L1 blockers, has been shown to amplify T cell-driven anti-tumor responses, resulting in enhanced tumor suppression (Xiong et al., 2021). Myeloid-derived suppressor cells (MDSCs) are immunosuppressive cells that accumulate under pathological conditions, inhibiting dendritic cell (DC) functions and T-cell immune responses. In colon cancer, targeting *ASAH2* on MDSCs infiltrating tumors with NC06 has been shown to induce ferroptosis in these cells, thereby reducing their presence in tumor cells. This reduction subsequently activates cytotoxic T lymphocytes, enhancing tumor suppression (Zhu et al., 2021). These findings suggest NC06’s potential as a therapeutic approach to modulate the pro-inflammatory immune tumor microenvironment. In the LAR subtype of TNBC, elevated GPX4 levels enhance anti-tumor immunity; however, inhibiting GPX4 induces ferroptosis, particularly when combined with anti-PD1 therapy, leading to greater efficacy compared to monotherapy. Conversely, in glioma and TNBC, GPX4 knockout in B cells suppresses immune responses through lipid peroxidation and ferroptosis (Jiang L. et al., 2023; Yang F. et al., 2023; Yang et al., 2014). Overall, immunotherapy in TNBC exhibits a dual effect influenced by ferroptosis, with these interactions shaped by tumor heterogeneity and the TME.

Given that copper metabolism and cuproptosis significantly influence tumor immunity, targeting cuproptosis may enhance immunotherapy effectiveness. The PD-1/PD-L1 pathway is a key immune checkpoint, and inhibiting PD-1/PD-L1 could improve clinical outcomes for cancer patients (Tiwari et al., 2022). Copper may positively regulate PD-L1 expression in tumors. For example, disulfiram–Cu enhances PD-L1 levels in hepatocellular carcinoma (HCC) cells by inhibiting PARP1 and promoting GSK-3β phosphorylation, which reduces T-cell infiltration. Targeting cuproptosis alongside PD-1/PD-L1 inhibitors could improve therapeutic outcomes. Preclinical studies demonstrate that combining copper ionophores with anti-PD-L1 agents enhances tumor growth suppression in pancreatic ductal adenocarcinoma (PDAC), lung cancer, and HCC (Huang et al., 2023; Li P. et al., 2023; Zhou et al., 2019). In the context of hormone therapy, which targets estrogen receptor-positive (ER+) breast cancers through agents like tamoxifen or aromatase inhibitors, resistance remains a challenge. Inducing ferroptosis in these hormone-dependent cancer cells could overcome resistance by targeting oxidative stress pathways that these cells exploit for survival. Additionally, recent studies suggest that endocrine therapies may increase ferroptosis sensitivity in HR+ cancers by potentially reducing GPX4 activity. The development of the FERscore (Hu et al., 2024), a ferroptosis susceptibility index, highlights a potential therapeutic avenue, suggesting that combining ferroptosis inducers with endocrine therapy could selectively target resistant tumor cells (Li P. et al., 2023). In terms of HER-2-positive breast cancer, ferroptosis holds the potential to overcome treatment resistance. Studies show that inhibiting FGFR4 (Zou et al., 2022) could induce ferroptosis in resistant cells, enhancing sensitivity to therapies like trastuzumab. Additionally, combining ferroptosis inducers with HER-2-targeted treatments boosts ROS production, leading to increased cell death. Neratinib, an irreversible HER-2 inhibitor, has been found to induce ferroptosis in HER-2-positive breast cancer models. By promoting mitochondrial dysfunction and increasing iron-dependent ROS levels, neratinib works synergistically with ferroptosis inducers like RSL3. This combination not only boosts cell death but also helps inhibit brain metastasis in HER-2-positive cases, indicating a dual advantage in targeting both HER-2 and ferroptosis pathways. The therapy pairing neratinib with RSL3 enhances lipid peroxidation and mitochondrial impairment, resulting in increased ferroptotic cell death, providing a promising strategy for improving outcomes in patients with HER-2-positive breast cancer who may not adequately respond to standard treatments (Hu et al., 2024; Park et al., 2023). Integrating ferroptosis-related biomarkers with tumor mutation burden evaluations could improve prognostic insights in HER-2-positive patients. This approach may help identify individuals who are more likely to respond positively to therapies that target ferroptosis in addition to standard anti-HER-2 treatments (Shi et al., 2024). Future research should focus on finding effective combinations of ferroptosis inducers with current HER-2-targeted therapies to improve treatment outcomes. Understanding the mechanisms that enhance efficacy is crucial. Additionally, ongoing clinical trials exploring the relationship between ferroptosis and anti-HER-2 therapies will be essential for validating findings and determining optimal strategies for treating HER-2-positive breast cancer patients.

Breast cancer cells demonstrate a distinct copper metabolism, requiring elevated copper levels, which makes them more vulnerable to cuproptosis-inducing agents. Research shows that disruptions in copper metabolism are closely linked to BRCA progression. Overexpression of copper transport proteins like SLC31A1 enhances copper uptake, leading to cuproptosis (Wang J. et al., 2024). Alterations in genes related to copper metabolism disrupt homeostasis, resulting in intracellular copper accumulation and triggering cell death through cuproptosis. Modulating the expression of these genes can effectively regulate BRCA cell growth and proliferation. For instance, TNBC is highly sensitive to cuproptosis inducers such as ZnPT, which interferes with copper balance, causing cell death by DLAT aggregation and inhibiting tumor proliferation, migration, and stemness (Yang X. et al., 2023). The interplay between cuproptosis and BRCA emphasizes the importance of molecular heterogeneity in therapeutic responses, with different BRCA subtypes showing varying sensitivity to cuproptosis-inducing treatments (Tang et al., 2024). Cuproptosis, driven by copper accumulation, impacts hormone receptor-positive (HR+) breast cancer prognosis. Elevated levels of genes like *DLAT, FDX1*, and *PDHA1* correlate with poor outcomes, greater tumor aggressiveness, and immune suppression. Treatment strategies could include copper modulation or combining cuproptosis inducers with hormone therapies. However, response variability across patients underlines the need for further research to refine personalized approaches (Li J. et al., 2022; Sha R. et al., 2024; Wang R. et al., 2023; Zhu Z. et al., 2024). Similarly, HER-2-positive BRCA cells, responsive to HER-2-targeted therapies like trastuzumab, warrant further investigation regarding their reaction to cuproptosis inducers (Sha R. et al., 2024). A recent study developed a prognostic model using four CRGs, identifying *DLAT* as an independent prognostic marker linked to resistance against HER-2-targeted therapy in HER-2-positive breast cancer patients. Combining HER-2-targeted treatments with cuproptosis inducers might enhance efficacy by affecting copper metabolism and HER-2 signaling pathways. TNBC, with its significant reliance on copper, presents a particularly promising target for cuproptosis-based therapies.

A promising avenue for advancing cancer therapy involves highlighting the interplay between ferroptosis and cuproptosis, aiming to target both pathways for improved therapeutic outcomes simultaneously. The close association between ferroptosis and cuproptosis with mitochondrial metabolism underscores the necessity of exploring mitochondria to fully grasp the underlying mechanisms, regulatory mechanisms, and implications for disease (Gao et al., 2024; Wang W. et al., 2023).

Moreover, GSH serves as a pivotal nexus for both ferroptosis and cuproptosis, in addition to its involvement in the mitochondrial TCA cycle. During ferroptosis, GSH functions as an antioxidant, impeding lipid peroxidation. In cuproptosis, GSH acts as a copper chaperone, binding copper to prevent the aggregation of lipoylated proteins. Intriguingly, GSH can suppress both ferroptosis and cuproptosis, indicating a reciprocal regulatory relationship in which GSH likely plays a pivotal role in facilitating communication between these processes (Liu and Chen, 2024; Tsui et al., 2024; Wang J. et al., 2024; Kciuk et al., 2024). Thoroughly comprehending the relationship and mutually influencing aspects of ferroptosis and cuproptosis in cancer should provide a theoretical foundation for synergic treatment approaches.

Moving forward, a crucial area for prospective investigation involves the development of signatures encompassing both ferroptosis and cuproptosis-related ncRNAs. A thorough understanding of the mechanisms underlying the genes associated with ferroptosis and cuproptosis identified in this review is paramount for further elucidation. Several limitations necessitate consideration concerning the ferroptosis and cuproptosis-related gene signatures. First, the studies primarily relied on public databases, inherently introducing selection bias into the study design. Second, despite certain key findings being substantiated by experimental validation, additional experiments are necessary to delve into potential mechanisms, particularly regarding the interaction between cuproptosis and ferroptosis. Evaluating the clinical efficacy of existing gene signatures will be critical for their possible integration into personalized treatment approaches for breast cancer.

Ferroptosis inducers can effectively kill cancer cells, but they may also harm immune cells, thereby suppressing anti-tumor responses. To address this issue, future research should focus on developing small-molecule drugs that selectively trigger ferroptosis in cancer cells while preserving immune function. In parallel, copper chelators and complexes have shown promise in enhancing chemotherapy and preventing resistance in preclinical studies; however, cuproptosis-targeted therapies remain underdeveloped compared to those for copper-related genetic diseases.

Co-targeting ferroptosis and cuproptosis by altering mitochondrial and metal ion homeostasis may personalize cancer treatment by utilizing tumor-specific iron and copper metabolic requirements. This approach aligns with the principles of precision medicine, potentially enhancing patient outcomes and treatment efficacy. Inducing both ferroptosis and cuproptosis can significantly boost the effectiveness of chemotherapy and immunotherapy. Additionally, while radiotherapy is known to trigger ferroptosis (Lei G. et al., 2020), its potential to induce cuproptosis remains unclear. Co-targeting these pathways shows promise over monotherapies, particularly within specific cancer types. However, further research is essential to uncover the underlying molecular mechanisms and address the potential damage to healthy tissues. Developing innovative therapies, especially nanomedicines, will be critical. A deeper understanding of the interactions between these regulated cell death pathways and breast cancer is necessary to enhance future therapeutic strategies.

## 8 Conclusion

This review highlights the dual-targeting of ferroptosis and cuproptosis as a transformative strategy for breast cancer therapy, focusing on pathways that activate both cell death processes to combat resistance, especially in challenging subtypes like TNBC. Current findings show that inducers of ferroptosis and cuproptosis can enhance sensitivity to traditional treatments, from chemotherapy to immunotherapy, and may address resistance in both advanced and early stages. However, effective clinical translation requires further investigation into tumor-specific iron and copper metabolism and strategies to mitigate potential toxicity to non-cancerous cells. These insights emphasize the potential for precision treatments tailored to exploit metabolic vulnerabilities, advancing the application of targeted therapies and novel combinational approaches for breast cancer management.

## Glossary

BRCA: breast cancer
Fe: iron
Cu: copper
DNA: deoxyribonucleic acid
ROS: reactive oxygen species
**Fe** ^**3+**^: ferric iron
**Fe** ^**2+**^: ferrous iron
STEAP3: six-transmembrane epithelial antigen of prostate 3
TFR1: transferrin receptor protein 1
MFRN: mitoferrin
FTH1: ferritin heavy chain 1
NCOA4: nuclear receptor coactivator 4
FPN: ferroportin
**Cu** ^**+**^: cuprous ion
**Cu** ^**2+**^: cupric ion
CTR1: copper transporter 1
Copper ATPases: copper-transporting P-type ATPases
BCSCs: breast cancer stem cells
DSF/Cu: disulfiram/copper
RAS-RAF-MEK-ERK: RAS-RAF-Mitogen-activated protein kinase kinase-extracellular signal-regulated kinase
RTK: receptor tyrosine kinase
TCA: tricarboxylic acid
DLAT: dihydrolipoamide S-acetyltransferase
TGN: trans-Golgi network
PRNP: prion protein
ATP: adenosine triphosphate
CISD1: CDGSH iron-sulfur domain-containing protein 1
CISD2: CDGSH iron-sulfur domain-containing protein 2
DBT: dihydrolipoyllysine-residue acetyltransferase component of pyruvate dehydrogenase complex
GCSH: glycine cleavage system H protein
DLST: dihydrolipoamide succinyltransferase
MCF: mitochondrial carrier family
SCD1: stearoyl-CoA Desaturase-1
DMOCPTL: dimethylaminomicheliolide
ncRNAs: non-coding RNAs
lncRNAs: long non-coding RNAs
miRNA: microRNA
circRNA: circular RNA
tsRNA: tRNA-derived small RNA
FRGs: ferroptosis-related genes
CRGs: cuproptosis-related genes
F/CRGs: ferroptosis/cuproptosis-related genes
ER+EBC: estrogen receptor-positive early breast cancer
iDFS: invasive disease-free survival
RFS: relapse-free survival
*GPX4*: glutathione peroxidase 4
*GSH*: glutathione
*ALDH*: aldehyde Dehydrogenase
*EMT*: epithelial-mesenchymal transition
*TNBC*: triple-Negative Breast Cancer
*DOX*: doxorubicin
*NPs*: nanoparticles
*CDT*: chemodynamic therapy
*SDT*: sonodynamic therapy
*PTT*: photothermal therapy
*ICD*: immunogenic cell death
*ECM*: extracellular matrix
*mtDNA*: mitochondrial DNA
LPO: lipid peroxidation.
